# Porcine Reproductive and Respiratory Syndrome Virus: Adaptive Immunity, Immune Evasion and Emerging Strategies for Disease Control

**DOI:** 10.3390/v18101077

**Published:** 2026-09-29

**Authors:** Ning Zhu, Jincheng Zhong, Zengjun Lu, Jing Zhang

**Affiliations:** 1 School of Biological and Pharmaceutical Engineering, Lanzhou Jiaotong University, Lanzhou 730070, China; 2 State Key Laboratory of Animal Disease Control and Prevention, College of Veterinary Medicine, Lanzhou University, Lanzhou Veterinary Research Institute, Chinese Academy of Agricultural Sciences, Lanzhou 730046, China; 3 Gansu Province Research Center for Basic Disciplines of Pathogen Biology, Lanzhou 730046, China

**Keywords:** PRRSV, adaptive immunity, immune evasion, vaccine, antiviral strategy

## Abstract

Porcine reproductive and respiratory syndrome virus (PRRSV) is one of the most economically important pathogens affecting the global swine industry. This review summarizes current knowledge of PRRSV-induced suppression of adaptive immune responses, with particular emphasis on impaired T cell activity, dysregulated B cell responses, delayed and insufficient neutralizing antibody production, and viral strategies that limit effective immune clearance. In addition, recent advances and limitations in vaccine development and antiviral strategies are presented. By integrating insights from viral pathogenesis and host immune responses, this review provides a comprehensive perspective on current challenges and emerging strategies for effective PRRSV control.

## 1. Introduction

Porcine reproductive and respiratory syndrome (PRRS) remains one of the most economically devastating infectious diseases affecting the global swine industry [1]. Clinically, the disease is characterized by severe reproductive failure in breeding herds, including late-term abortion, stillbirth, fetal mummification and the delivery of weak-born piglets, together with respiratory disease in growing pigs manifested by interstitial pneumonia, growth retardation, increased mortality and enhanced susceptibility to secondary bacterial infections [2,3].

The etiological agent, porcine reproductive and respiratory syndrome virus (PRRSV), is an enveloped, positive-sense, single-stranded RNA virus belonging to the family *Arteriviridae* within the order *Nidovirales* [4]. The International Committee on Taxonomy of Viruses has recently updated PRRSV taxonomy, dividing the virus into two distinct species based on genetic divergence. Under this new classification, PRRSV-1 and PRRSV-2 are designated as *Betaarterivirus europensis* and *Betaarterivirus americense* (previously *Betaarterivirus suid* 1 and *suid* 2), respectively. PRRSV-1 and PRRSV-2 exhibit substantial genetic divergence, with only approximately 50–70% nucleotide identity across their genomes [5]. Furthermore, each species contains multiple genetic lineages. A refined classification system based on 10, 446 global PRRSV-1 ORF5 sequences has recently categorized PRRSV-1 into four major lineages (L1–L4), with lineage 1 further divided into 18 sublineages (L1.1–L1.18) [6]. Based on global ORF5 sequence analyses, PRRSV-2 has been classified into 11 major genetic lineages (L1–L11) and 21 sublineages [7]. The prevalence of individual lineages varies markedly among countries and over time. For example, multiple PRRSV-2 lineages, including L1, L3, L5, and L8, have circulated in China, with lineage replacement and shifts in dominant sublineages occurring in recent decades [7]. These lineage-based classification systems facilitate the tracking of viral evolution, characterization of antigenic diversity, and assessment of the genetic relationship between circulating strains and commercial vaccine viruses. Owing to its extensive genetic diversity, marked capacity for immune evasion and ability to establish prolonged infection, PRRSV continues to present major challenges to disease surveillance, vaccine development, and effective control, thereby remaining a promising research direction in contemporary swine health and veterinary virology research [8,9].

Over the past three decades, substantial progress has been made in understanding PRRSV molecular biology, host-pathogen interactions and pathogenesis [10,11,12]. Despite extensive research, translating these insights into consistently effective PRRSV control under field conditions has not yet been fully accomplished [13]. A central obstacle is that PRRSV elicits immune responses that are often delayed, incomplete or functionally dysregulated [14]. The slow development of neutralizing antibodies, the limited protective T cell responses and the capacity of the virus to subvert both innate and adaptive immunity have complicated the identification of robust protection [15]. A better understanding of the immune mechanisms underlying PRRSV infection is essential for vaccine development [16,17].

This review summarizes current understanding of PRRSV adaptive immunity and immune evasion, with particular emphasis on the interplay between humoral and cellular immune responses. It further examines the strengths and limitations of existing vaccine platforms, emerging next-generation vaccine strategies and recent progress in antiviral strategy development. Collectively, these areas provide an integrated framework for understanding why PRRSV remains difficult to control and which directions are most likely to shape future intervention strategies.

## 2. Genome Organization and Protein Functions of PRRSV

**Figure 1:**
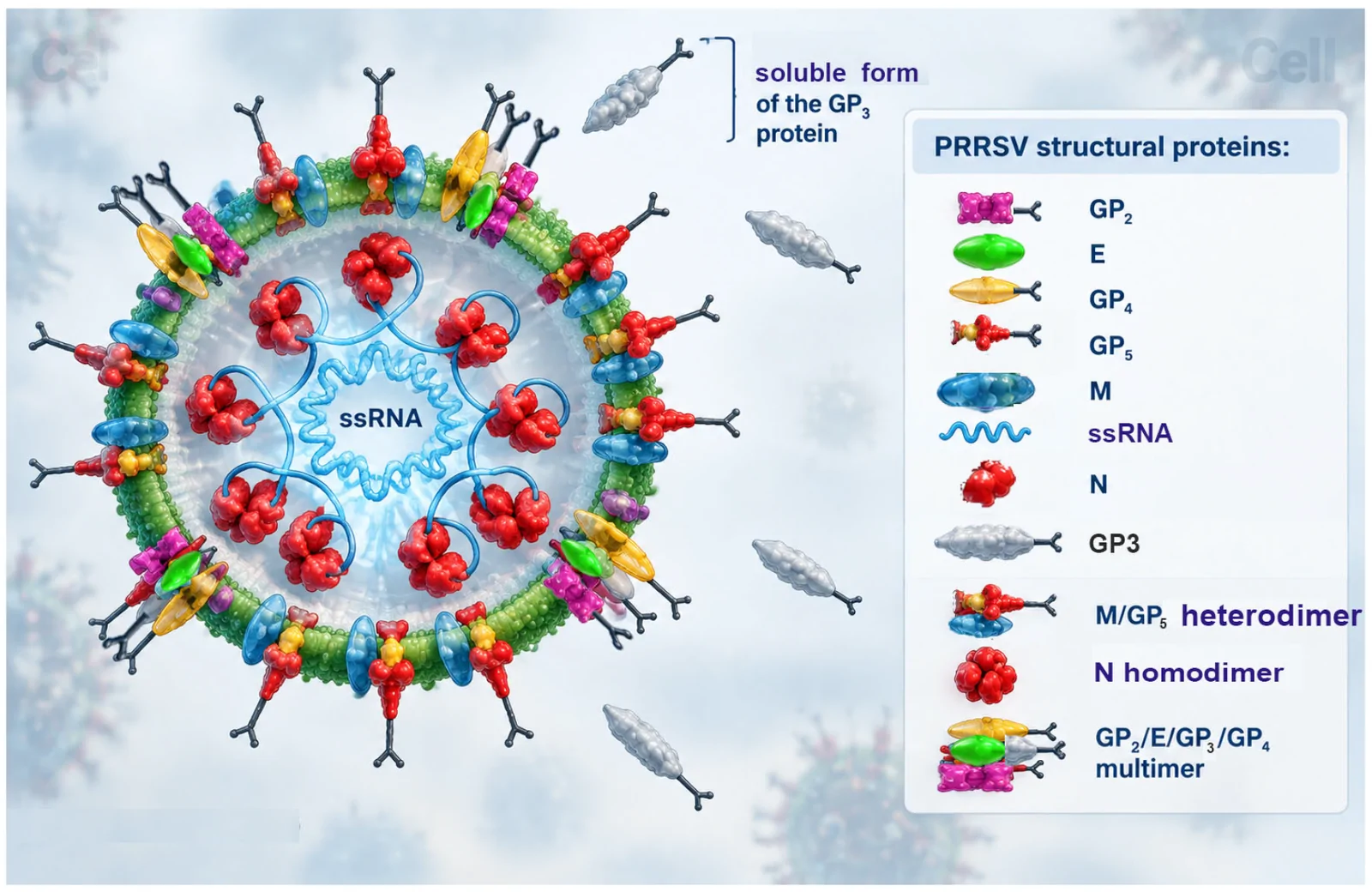
The porcine reproductive and respiratory syndrome virus (PRRSV) virion and its structural protein components. PRRSV is an enveloped positive-sense single-stranded RNA (ssRNA) virus. The viral envelope contains multiple membrane-associated structural proteins, including the M/GP_5_ heterodimer and the GP_2_/E/GP_3_/GP_4_ multimeric complex. The nucleocapsid, assembled from N protein homodimers, encapsidates the viral ssRNA genome. A soluble form of GP_3_ protein is also illustrated as a secreted product separate from intact virions or G2/E/G3/G4 complex. The legend box summarizes the identity and oligomeric assembly of all depicted PRRSV structural proteins. This figure was created in BioRender https://BioRender.com/vqylla5. Agreement number CL2A90ZLFQ. This figure has been modified by GPT-image2.5.

The PRRSV genome is approximately 15–15.5 kb in length and contains at least 11 open reading frames (ORFs). ORF1a and ORF1b occupy nearly three-quarters of the genome and encode the replicase polyproteins pp1a and pp1ab, which are subsequently processed into multiple nonstructural proteins (nsps) [18]. In addition, a trans-frame ORF (TF ORF) embedded within ORF1a is expressed via programmed -2 ribosomal frameshifting and encodes the nonstructural protein NSP2TF. The nonstructural proteins are generated by proteolytic cleavage of the replicase polyproteins pp1a and pp1ab [19]. They primarily function in viral RNA synthesis and antagonism of innate immunity. These include proteases such as nsp1α, nsp1β, nsp2, and nsp4, as well as replication-associated proteins such as nsp9 (RNA-dependent RNA polymerase), nsp10 (helicase), and nsp11 (endoribonuclease) [20,21,22]. In addition to orchestrating the formation of the replication and transcription complex, several nsps, particularly nsp1, nsp2, and nsp11, have been shown to interfere with interferon production and other antiviral signaling pathways, thereby facilitating viral persistence and pathogenesis [23,24,25,26,27,28]. The downstream region encodes the structural proteins through ORF2a, ORF2b, ORF3–ORF4, ORF5a, and ORF5–ORF7 [19]. These structural proteins include the major envelope glycoprotein GP5, the membrane protein M, the nucleocapsid protein N, and the minor envelope proteins GP2a, E (GP2b), GP3, GP4 and ORF5a [29,30,31]. Among these, GP5 and M form heterodimers essential for virion assembly and infectivity, while the minor glycoproteins are involved in receptor interaction and viral entry [29,30,31]. The structural organization of PRRSV virions is illustrated in Figure 1.

## 3. Adaptive Immunity to PRRSV

### 3.1. Humoral Immunity and B Cell Response

B cells are central to antiviral immunity because they provide both immediate humoral protection and long-term immune memory [32]. Upon antigen encounter, activated B cells differentiate into antibody-secreting plasma cells and memory B cells, thereby contributing to both early control of infection and accelerated memory B cell responses upon re-exposure [33]. Both PRRSV-1 and PRRSV-2 infections induce early virus-specific antibody responses; however, functional neutralizing antibodies generally develop only at later stages of infection, typically several weeks after exposure [34,35,36].

In PRRSV-2 infection models, serum viral loads commonly peak around 7–10 dpi and may persist for several weeks, whereas PRRSV-1 infection shows similar kinetics, with peak viremia generally occurring within the first 1–2 weeks after infection [37]. Both PRRSV-1 and PRRSV-2 infections induce early virus-specific antibody responses; however, the development of protective humoral immunity is delayed [38]. The early antibody response is dominated by IgM, which is transient, peaking between 14 and 21 dpi and declining to undetectable levels around 40 dpi [39]. In contrast, the IgG response rises more slowly, peaking at 21–28 dpi, and remaining elevated during the persistent phase of infection [40]. Serologically, antibodies against the nucleocapsid (N) protein are among the earliest and most prominent antibody responses detected following PRRSV infection [39]. However, these antibodies produced in the early stage do not confer protective effects and lack neutralizing activity [35,41]. By contrast, virus-neutralizing antibodies generally emerge only later, most often around 28 dpi, consistent with the delayed development of protective humoral immunity during PRRSV infection [39,40,41]. Together, these observations indicate that although B cell activation occurs relatively early, the generation of functionally protective antibody responses is substantially delayed [35,36,38,41,42,43].

Despite their delayed appearance, passive immunization studies have provided compelling evidence for protective efficacy against both PRRSV-1 and PRRSV-2 infection. In PRRSV-2 challenge models, administration of PRRSV-neutralizing immunoglobulins protected pregnant sows from reproductive failure, prevented vertical transmission, and resulted in clearance of infectious virus from maternal tissues [44,45]. More recently, a study using broadly neutralizing antibodies demonstrated protective effects against both PRRSV-1 and PRRSV-2 challenges, further confirming the cross-protective potential of functional neutralizing antibodies [46]. Accordingly, considerable efforts have been devoted to identifying the viral proteins and epitopes that elicit protective neutralizing antibody responses.

Early studies initially regarded GP5 as the principal neutralization target [47]. A conserved neutralization-associated region was identified in the GP5 ectodomain, with a core spanning amino acids (a.a.) 37 to 45, but subsequent work suggested that GP5-mediated neutralization cannot be explained merely by a simple linear epitope and instead depends highly on native three-dimensional conformation of the protein [47]. This interpretation is supported by vaccine studies showing that both PRRSV-1 and PRRSV-2 GP5-based immunogens expressed alone or in combination with M (membrane) protein can induce neutralizing antibodies, particularly when presented in a structurally relevant context [48,49]. The M protein, which heterodimerizes with GP5, likely contributes to the formation and stabilization of the conformational neutralizing surface [50,51]. Consistent with this view, reverse genetics studies have identified residue 70 in M, and residues 102 and 104 in GP5 as important determinants of PRRSV-2 susceptibility to serum neutralization [51].

**Figure 2:**
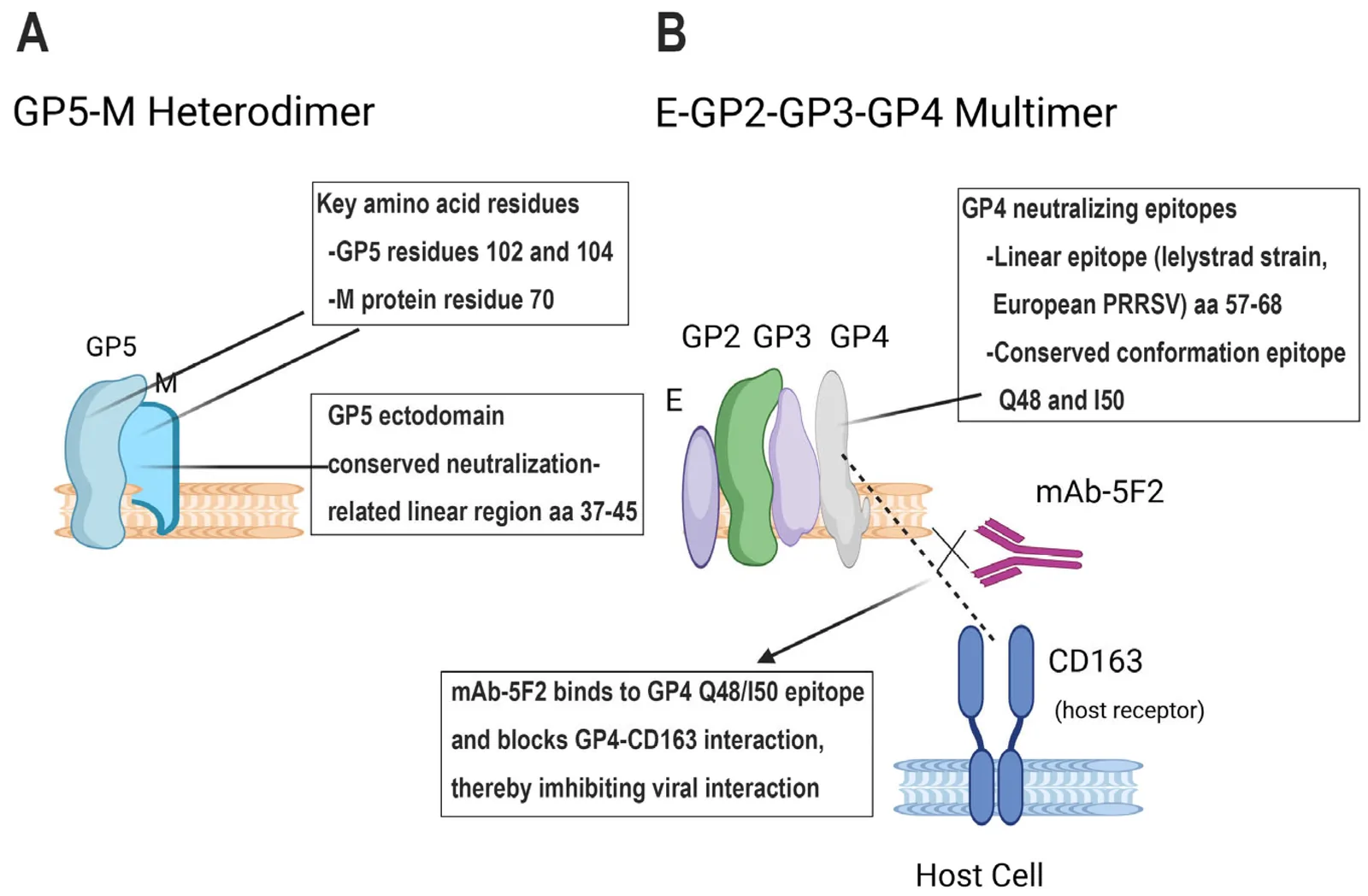
Antigenic epitope targets of PRRSV neutralizing antibodies. An illustration of major neutralizing antibody targets on the PRRSV virion. (**A**), The GP5-M heterodimer contains key neutralizing epitopes, including the conserved GP5 ectodomain linear region (aa 37–45) and conformational determinants associated with GP5 residues 102 and 104. The M protein contributes to the stabilization of GP5 conformational epitopes through critical residue M70. (**B**), The GP2/GP3/GP4 complex mediates host receptor recognition and viral entry. GP4 harbors neutralizing epitopes, including the Lelystad strain linear epitope (aa 57–68) and conserved conformational residues Q48 and I50. The monoclonal antibody mAb-5F2 targeting the GP4 Q48/I50 epitope blocks GP4-CD163 interaction and inhibits viral internalization. This figure was created in BioRender, https://BioRender.com/9nj2ete. Agreement number RL2A90KCKU.

More recently, attention has raised toward the minor glycoprotein complex GP2/GP3/GP4, which is recognized as being directly involved in receptor engagement and viral entry, there by highlighting these proteins as important targets of antibody-mediated neutralization [19,52,53]. Indeed, both linear and conformational neutralizing determinants have been identified within this complex, particularly on GP4, and at least some broadly neutralizing activity appears to be associated with interference in receptor binding [54]. The GP4 protein of European genotype PRRSV Lelystad strain contains a highly immunogenic linear epitope (a.a. 57–68) that induces neutralizing antibodies [51]. More recently, a broadly neutralizing monoclonal antibody, mAb-5F2, was isolated using hybridoma technology and demonstrated cross-lineage neutralization against both PRRSV-1 strains and PRRSV-2 strains belonging to lineages 1, 5, and 8 [55]. This antibody recognizes a conformational epitope on the GP4 protein, targeting the conserved residues Q48 and I50 [52]. Mechanistically, mAb-5F2 inhibits infection by blocking the GP4-CD163 interaction, thereby preventing viral internalization [56]. More recently, structural studies using PRRSV-2 identified another GP4 neutralizing epitope located within the N-terminal region (a.a. 59–69), with S60, E61, and K65 serving as critical antibody-contact residues. Although this monoclonal antibody recognizes a structurally relevant GP4 surface and efficiently neutralizes the homologous PRRSV-2 VR-2332 strain, it fails to neutralize divergent PRRSV-1 and PRRSV-2 strains, highlighting the strain-specific nature of GP4-mediated neutralization [57]. Beside this, the major antigenic determinants recognized by PRRSV-neutralizing antibodies are demonstrated in Figure 2. Taken together, current evidence suggests that PRRSV neutralization is mainly mediated by structurally complex antigenic determinants distributed across multiple envelope proteins [47,56].

### 3.2. Viral Evasion of Humoral Immunity

**Figure 3:**
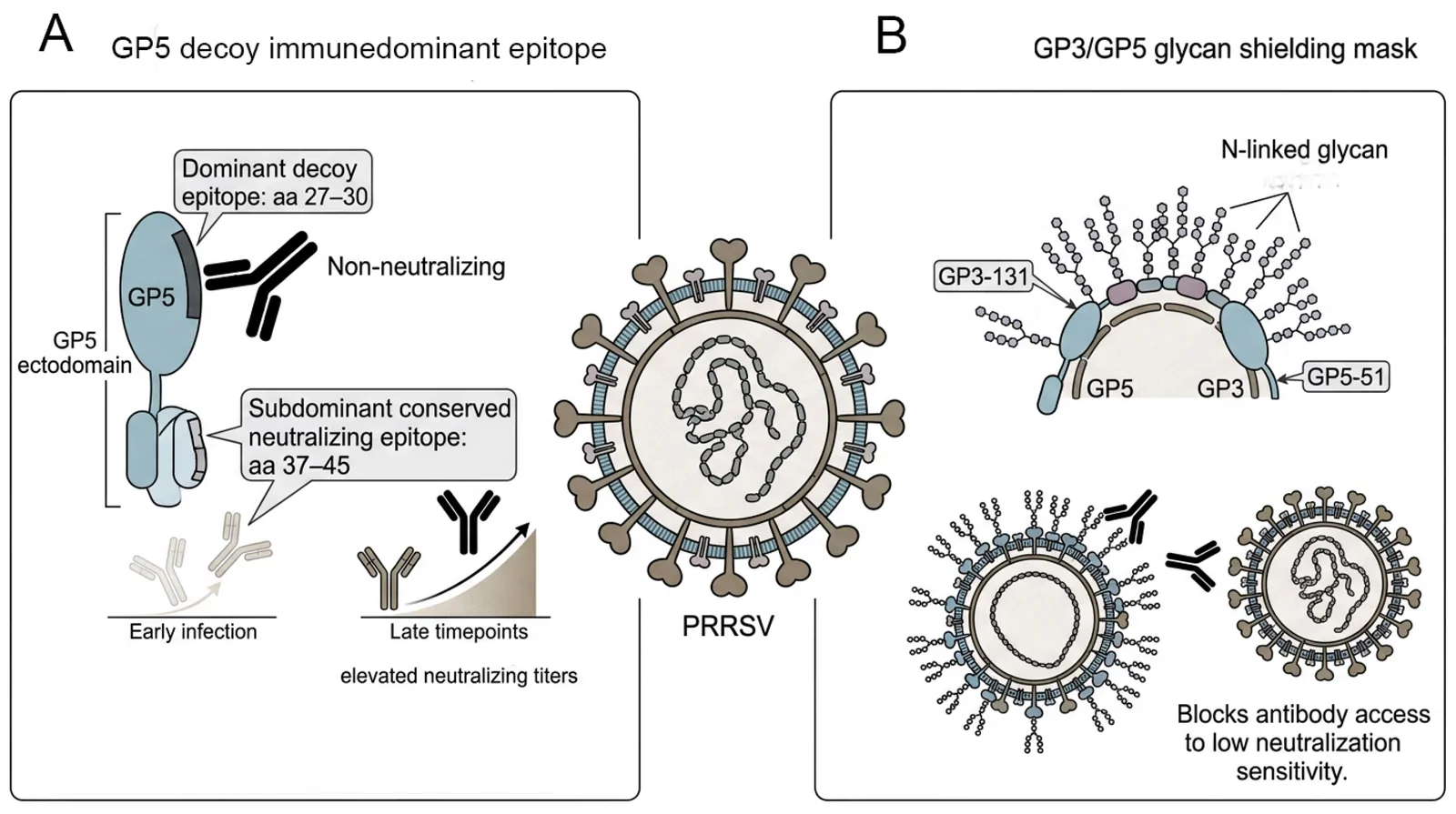
PRRSV immune evasion mechanisms that delay protective neutralizing antibody responses. An overview of three strategies used by PRRSV to impair effective adaptive immunity. (**A**), PRRSV GP5 exploits epitope hierarchy by diverting early antibody responses toward a dominant non-neutralizing decoy epitope (aa 27–30), while delaying responses against the conserved neutralizing epitope (aa 37–45). (**B**), Glycan shielding mediated by GP3-131 and GP5-51 N-linked glycans masks neutralizing epitopes and reduces antibody accessibility, whereas glycan-deficient viruses exhibit enhanced neutralization sensitivity and accelerated neutralizing antibody induction. This figure was created in BioRender https://BioRender.com/6i37qzn. Agreement number SC2A90UTL4.

Although neutralizing antibodies can be protective, PRRSV has evolved several strategies to evade or delay effective humoral immunity (Figure 3). At the viral antigenic level, PRRSV can manipulate antibody responses by directing B cell recognition toward non-protective epitopes [47,58]. Ostrowski and colleagues demonstrated that PRRSV-2 infection preferentially elicits an early, immunodominant non-neutralizing “decoy” epitope in the GP5 ectodomain (approximately residues 27–30), whereas a more conserved neutralization-associated epitope (approximately residues 37–45) remains subdominant and is effectively targeted only later, coincident with rising neutralizing titers [36]. In addition to epitope hierarchy, PRRSV can reduce antibody access to key epitopes through glycan shielding. Evidence suggests that both GP5 and GP3 contribute to a glycan-based mask that limits the exposure of key neutralizing epitopes [58]. A field isolate of PRRSV-2 lacking two N-glycosylation sites (GP3-131 and GP5-51) was unusually sensitive to heterologous sera and induced higher neutralizing antibody titers earlier in pigs [58]. Using reverse genetics, restoring either glycosylation site reduced neutralization sensitivity, and restoring both produced shielding effect [58]. Notably, viruses in vivo rapidly evolved increased neutralization resistance, consistent with selection pressure to establish glycan shielding [58]. Together, these findings support a GP3/GP5 glycan “mask” that limits epitope accessibility and delays the development of effective neutralizing antibody responses [58].

Beyond direct antigenic evasion, PRRSV can modulate host immune pathways involved in both innate antiviral defense and the subsequent development of protective adaptive immunity [59,60]. PRRSV primarily infects porcine alveolar macrophages (PAMs) and monocytes, which play important roles in initiating antiviral immune responses [61]. Viral proteins can interfere with host signaling pathways by suppressing interferon-α/-β (IFN-α/-β) production, promoting IL-10 expression, and altering antigen presentation processes, thereby creating an immunosuppressive environment that may impair effective antiviral immunity [62,63,64,65]. Moreover, both PRRSV-1 and PRRSV-2 infections have been shown to induce immunoregulatory cytokines, especially IL-10. Increased IL-10 production has been associated with reduced antiviral T cell responses and impaired B cell activation and antibody maturation, which may contribute to delayed and suboptimal neutralizing antibody responses [9,65,66,67,68,69,70]. A further and more indirect mechanism may involve impairment of T cell help. Because CD4^+^ T cells are essential for optimal B cell activation, affinity maturation, and class switching, PRRSV-induced disruption of thymic development and peripheral CD4^+^ T cell production may weaken the quality of humoral immunity [71]. Collectively, the delayed appearance and limited potency of PRRSV-specific neutralizing antibodies likely result from a combination of viral antigenic escape mechanisms and broader disruption of adaptive immune coordination.

### 3.3. T Cell Responses

Although neutralizing antibodies have long been a major focus of PRRSV immunology, accumulating evidence indicates that pigs are capable of controlling infection even in the absence of detectable neutralizing antibodies [36,41,72]. Conversely, viremia may persist despite the presence of neutralizing antibodies [73,74]. Together, these findings suggest that additional immune mechanisms contribute substantially to viral control and clearance during PRRSV infection. While some of these effects may reflect the non-neutralizing activities of antibodies, the contribution of T cell-mediated immunity remains incompletely defined and warrants further investigation [12].

T cell responses to PRRSV are generally delayed and tissue-specific, and fail to develop into rapid and coordinated protective immunity [9]. According to the T cell receptor (TCR) chains they express, T cells are broadly divided into αβ and γδ T cells [75,76]. Within the αβ T cell compartment, the major subsets are conventionally defined by co-receptor expression as CD4^+^ and CD8^+^ T cells [77]. Notably, in contrast to humans and mice, pigs possess a relatively high frequency of peripheral and secondary lymphoid CD4^+^CD8^+^double-positive (DP) T cells, highlighting species-specific features of porcine T cell biology that should be considered when interpreting antiviral immune responses [78,79]. γδ T cells are a distinct subset of T lymphocytes characterized by expression of the γδ T cell receptor (TCR) [80]. In humans, mice, and dogs, they represent only a minor proportion of circulating T cells (approximately 5%). In contrast, under physiological conditions, γδ T cells can account for more than 50% of the circulating T cell population in pigs, cattle, and chickens [81,82].

During both PRRSV-1 and PRRSV-2 infection, T cell dynamics are characterized by an early phase of variable peripheral T cell changes followed by the delayed development of virus-specific cellular immunity. In PRRSV-1 infection, transient decreases in circulating CD4^+^ T cells have been reported during the acute phase, followed by recovery toward baseline and increased recruitment of CD8^+^ T cells to the respiratory tract [83,84]. Virus-specific proliferative responses generally become evident only after several weeks, whereas functional cytotoxic responses can remain weak or markedly delayed [83,84]. PRRSV-2 infection shows a broadly similar pattern, although early reductions in circulating CD4^+^ and/or CD8^+^ T cells are particularly evident with some highly pathogenic strains and are not consistently observed across all isolates [85,86,87,88]. PRRSV-2-specific proliferative and interferon-gamma (IFN-γ)-producing T cell responses typically become detectable from approximately 2 weeks post-infection and increase substantially around 21–28 dpi [85,86]. Thus, although early changes in circulating T cell populations are highly strain-dependent, delayed and tissue-dependent development of virus-specific T cell immunity appears to be a shared feature of PRRSV-1 and PRRSV-2 infection [41,89,90].

#### 3.3.1. CD4^+^ T Cell Subsets

Within the CD4^+^ T cell population, several functional subsets have been described, including Th1, Th2, Th17, follicular helper T (Tfh) and regulatory T (Treg) cells [91]. Available evidence indicates that both PRRSV-1 and PRRSV-2 can induce Th1-associated responses, although their magnitude and kinetics vary among viral strains and experimental models. Th1 cells are driven by IL-12, IFN-α/-β, and IFN-γ, and are characterized by expression of the transcription factor T-bet and production of IFN-γ and TNF-α [90,92]. In pigs, IFN-γ production is commonly used as a functional indicator for Th1-like antiviral CD4^+^ T cell responses [12,86]. Consistent with this concept, PRRSV-1 infection has been associated with increased T-bet expression in peripheral blood CD4^+^ T cells, and PRRSV-restimulated PBMCs from convalescent pigs exhibit a clear Th1-type cytokine profile, most notably marked induction of IFN-γ mRNA [93,94]. Similarly, the development of a Th1-associated CD4^+^ T cell response has also been observed during PRRSV-2 infection, with T-helper cells representing the major IFN-γ -secreting lymphocyte population [85]. Together, these findings indicate that Th1 polarization is a prominent feature of the adaptive response to PRRSV and may contribute to viral control.

Th17 cells, characterized by RORγt expression and production of IL-17 and IL-22, are important for immunity against extracellular bacteria and fungi [95]. In the setting of PRRSV infection, impaired Th17 responses have been reported, particularly following infection with highly pathogenic PRRSV strains [96]. Notably, piglets inoculated with a Chinese highly pathogenic PRRSV strain showed reduced Th17 responses in peripheral blood and lung tissue, accompanied by increased bacterial burdens in the lungs compared with animals infected with a low-pathogenic PRRSV strain [96]. These findings suggest that suppression of the Th17 axis may contribute to increased susceptibility to secondary bacterial infection during severe PRRSV disease.

Tfh cells are typically defined by expression of CXCR5, PD-1, and ICOS, together with the lineage-specifying transcription factor Bcl-6 and production of IL-21 [97]. Tfh cells represent another CD4^+^ T cell subset of particular relevance to PRRSV, given their essential role in supporting germinal center reactions, B cell maturation, and the generation of antibody-secreting cells and memory B cells [98]. Evidence linking Tfh responses to PRRSV immunity is still emerging; however, a recent study using a chimeric PRRSV-1 expressing consensus ORF2-6 sequences showed enhanced heterologous neutralizing antibody responses accompanied by an increased proportion of Tfh cells in the tracheobronchial lymph nodes [98]. Similarly, immunization with the attenuated NADC34-like PRRSV-2 strain elicited Tfh responses together with neutralizing antibodies and PRRSV-specific IFN-γ -secreting cells, which were associated with protection against heterologous NADC34-like PRRSV-2 challenge [99]. Conversely, single-cell transcriptomic analysis suggested that highly pathogenic PRRSV may compromise humoral immunity by impairing Tfh cell function rather than reducing Tfh cell abundance, as defective Tfh activation and disrupted Tfh-germinal center B cell crosstalk were associated with impaired neutralizing antibody production [100].

Treg responses have also attracted considerable attention in PRRSV research because of their potential to suppress antiviral immunity. Tregs suppress effector responses through multiple mechanisms, including secretion of inhibitory cytokines such as TGF-β and IL-10, modulation of antigen-presenting cell (APC) function, and induction of effective T cell apoptosis [101,102]. In pigs, two major Treg subsets have been described: CD4^+^CD8^−^CD25^+^Foxp3^+^ and CD4^+^CD8^+^CD25^+^Foxp3^+^ cells [72,103,104,105]. Evidence from both in vitro and in vivo studies suggests that PRRSV can modulate Treg responses, although the outcome appears to depend on viral genotype, strain, and experimental context [105]. In vitro, PRRSV-2-infected dendritic cells (DCs) were shown to induce Foxp3^+^CD25^+^ T cells in co-cultured lymphocytes, and this effect was primarily dependent on TGF-β rather than IL-10 [106]. By contrast, PRRSV-1-infected DCs, despite producing high levels of IL-10, failed to induce Treg differentiation in co-cultured lymphocytes [107]. These observations indicate that PRRSV-mediated Treg induction is not solely determined by IL-10 production and may differ substantially between PRRSV genotypes [107]. In vivo studies further support Treg induction during both PRRSV-1 and PRRSV-2 infection, as increased Treg frequencies have been reported in blood, lungs, and lymphoid tissues, and in some studies the expansion of Tregs positively correlated with viremia [89,105,108,109]. Together, these findings suggest that PRRSV-induced Treg responses may contribute to immune suppression and viral persistence.

#### 3.3.2. Cytotoxic T Cell Responses

Cytotoxic T lymphocyte (CTL) responses are generally regarded as a key arm of antiviral immunity; however, in the context of PRRSV infection, these responses are often weak, delayed, and functionally heterogeneous [110,111]. Using PBMCs from pigs infected with the PRRSV Lelystad virus, PRRSV-specific CTL activity against infected alveolar macrophages was not detectable in freshly isolated PBMCs for up to 56 dpi [110]. Even after in vitro re-stimulation with PRRSV, although proliferation of CD3^+^CD8^high^ cells became apparent from 14 dpi onward, cytolytic activity remained largely absent and was only weakly detected in one animal at 56 dpi [110]. In the same experimental setting, pigs infected with pseudorabies virus developed robust SLA-I-restricted CTL responses by 21 dpi, highlighting the unusually poor induction of CTL activity by PRRSV. This impaired cytotoxic response was also accompanied by lower IFN-γ secretion after PRRSV re-stimulation, together suggesting functional impairment of PRRSV-specific cell-mediated immune responses [110]. Importantly, an in vitro study showed that cytotoxic T cell responses capable of killing PRRSV-2 infected cells have been detected in PAMs derived from pigs that recovered from PRRSV infection, supporting a role for cell-mediated cytotoxicity in protective immunity [100]. PBMCs from PRRSV-recovered pigs were shown to eliminate infected macrophages through a biphasic granzyme B-mediated response [112,113]. In this setting, an early killing phase (3-6 hpi) was mediated by CD4^+^CD8^high^ T cells, whereas a later phase (16-24 hpi) was mediated exclusively by CD4^+^CD8^−^ T cells [112]. Given that these pigs displayed only very low neutralizing antibody titers (1:4–1:16), these findings suggest that T cell-mediated immunity may make a substantial contribution to viral clearance after infection has been controlled [112].The contribution of individual T cell subsets to protective immunity against PRRSV appears to depend on both viral genotype and disease context. Heterologous stimulation with highly pathogenic lineage 8.7 PRRSV-2 tends to induce stronger IFN-γ responses in CD8^+^ than in CD4^+^ T cells. Conversely, protection against PRRSV-1-induced reproductive disease has been more closely associated with CD4^+^ T cell responses, with a comparatively limited contribution from CD8^+^ T cells [112,113]. Taken together, the available data indicate that PRRSV-specific cytotoxic responses are not absent, but rather delayed and functionally constrained during acute infection.

#### 3.3.3. γδ T Cells

Both PRRSV-1 and PRRSV-2 infections have been shown to induce γδ T cell proliferation; however, a direct role for these cells in PRRSV-specific cytotoxicity has not been established [84,105]. An in vivo study showed that PRRSV-2 infection induced a sustained γδ T cell response [114]. The number of circulating γδ T cells increased significantly from day 14 to day 70 post-infection [114]. Following antigen stimulation, γδ T cells began to proliferate at day 14 post-infection, and this proliferative response persisted until day 60 [114]. In addition, γδ T cells started to produce IFN-γ at day 14 post-infection, and this response continued until day 50 [114]. Upon re-exposure to PRRSV, both γδ T cells and CD4^+^ T cells showed increased IFN-γ production [114]. Together, these findings indicate that γδ T cells actively respond to PRRSV infection and may contribute to antiviral immunity [115]. Porcine γδ T cells can exhibit cytotoxicity in a cell contact- and degranulation-dependent manner in vitro [83]. However, they did not show specific cytotoxicity toward PRRSV-2 infected macrophages compared with mock-treated macrophages in one experimental study [83]. Therefore, the role of γδ T cells in controlling PRRSV infection remains unclear [83].

### 3.4. Mechanisms of T Cell Immune Evasion

**Figure 4:**
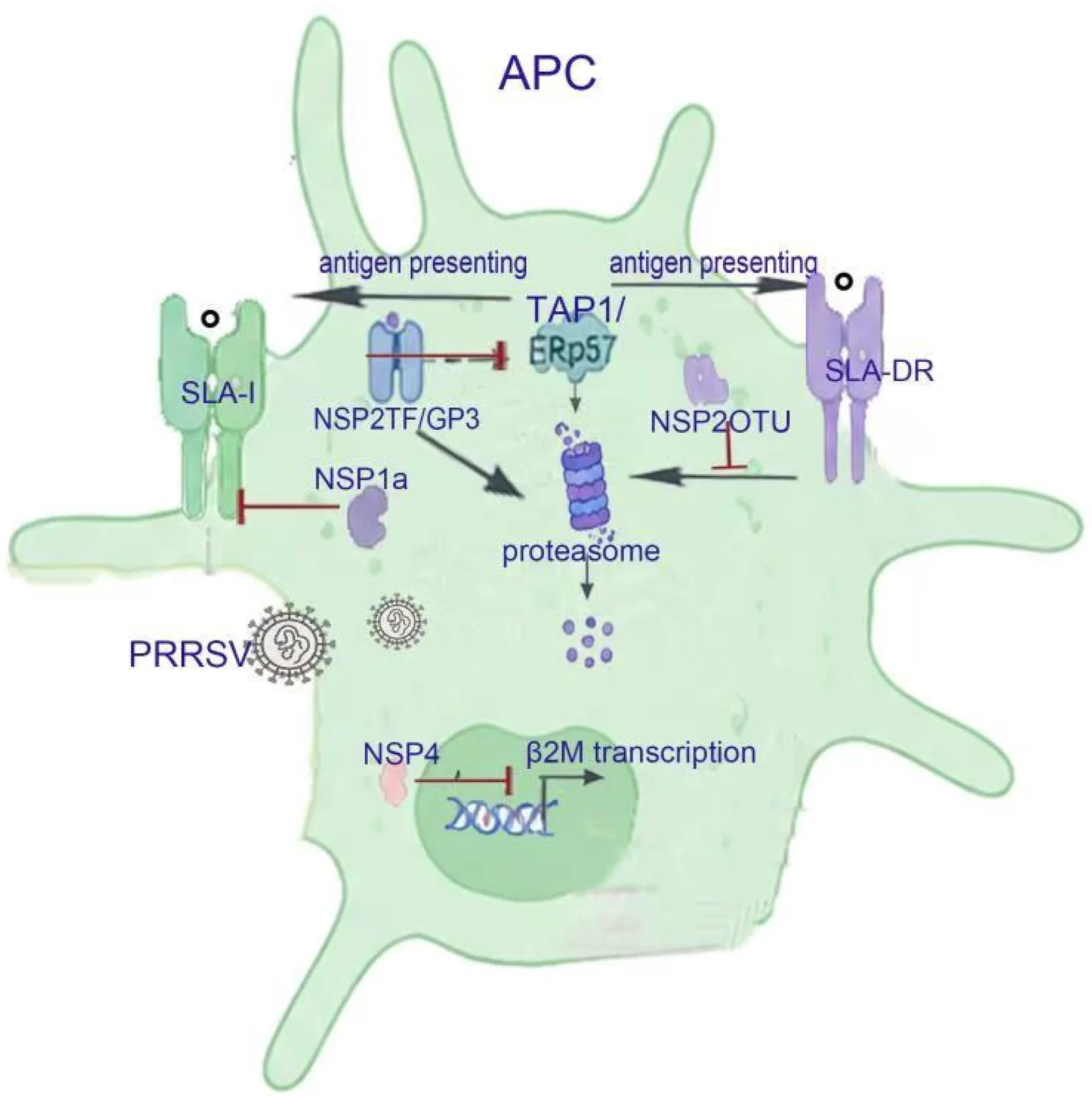
PRRSV-mediated disruption of antigen presentation. An overview of three proposed mechanisms underlying PRRSV-induced adaptive immune suppression. PRRSV impairs SLA-I antigen presentation through viral proteins such as nsp1α, nsp2TF, and GP3, with nsp4-mediated inhibition of β2-microglobulin (B2M) transcription and reduced TAP1/ERp57 expression contributing to defective CD8^+^ T cell activation. PRRSV selectively remodels the SLA-II pathway, where the nsp2 OTU domain stabilizes SLA-DR through deubiquitination and alters antigen presentation, promoting non-protective immune responses. This figure was created in BioRender https://BioRender.com/hjtsf57. Agreement number GM2A9122BL.

PRRSV suppresses T cell-mediated immunity through multiple, interconnected mechanisms that collectively impair antiviral cellular responses and promote viral persistence [9,15]. Broadly, these mechanisms include impairment of antigen presentation, attenuation of effective effector T cell activation and function, induction of immunosuppressive regulatory T cell (Treg) responses and upregulation of immune checkpoint molecules and promotion of T cell exhaustion [9,15,115] (Figure 4).

#### 3.4.1. Impaired Antigen Presentation

The functional limitations of PRRSV-specific T cell responses may, at least in part, originate upstream at the level of antigen presentation. Effective T cell activation depends on the presentation of antigenic peptides by major histocompatibility complex (MHC) class I and class II molecules [116]. In pigs, this is referred to as the swine leukocyte antigen (SLA) region [117]. Endogenous antigens are typically presented via SLA class I to CD8^+^ T cells, whereas exogenous antigens are presented via SLA class II to CD4^+^ T cells [118]. Therefore, interference with SLA-dependent antigen presentation represents a mechanism by which PRRSV may impair both priming and effector phases of adaptive immunity [9,60,77,115,119,120].

Several studies have shown that both PRRSV-1 and PRRSV-2 suppress the SLA-I pathway in porcine APCs [121,122]. Screening of PRRSV-2 structural and nonstructural proteins identified nsp1α, nsp2TF, and GP3 as viral proteins capable of significantly reducing SLA-I expression, with nsp2TF exerting the strongest effect [123]. In addition, nsp1α has been shown to downregulate SLA-I in porcine alveolar macrophages by targeting SLA-I molecules for proteasomal degradation [124]. PRRSV-2 also impairs other components of the antigen-processing machinery. In highly pathogenic PRRSV infection, nsp4 suppresses transcription of β2-microglobulin (β2M), a critical component of the SLA-I complex, thereby reducing intracellular β2M levels and weakening CD8^+^ T cell activation [125]. This suppression was found to be mediated by the viral nonstructural protein 4 (Nsp4), which binds to the porcine *B2M* promoter and inhibits its transcriptional activity [125]. Moreover, PRRSV-2-infected monocyte-derived DCs display reduced expression of TAP1 and ERp57, together with diminished T cell stimulatory capacity and reduced CD83 up-regulation, suggesting broader disruption of antigen processing and peptide loading [126].

Interestingly, PRRSV appears to regulate the SLA-II pathway differently from the SLA-I pathway. The swine leukocyte antigen DR (SLA-DR) protein complex is a transmembrane heterodimer composed of an α chain of approximately 34 kD and a β chain of approximately 29 kD [118]. In either PRRSV-1 or PRRSV-2 infected bone marrow-derived DCs and porcine alveolar macrophages, the protein level of the SLA-II molecule SLA-DR is increased despite a marked decrease in its mRNA [127]. Mechanistically, this discrepancy has been attributed to the deubiquitinating activity of the ovarian tumor (OTU) domain of PRRSV nsp2, which stabilizes SLA-DR by counteracting its ubiquitin-mediated degradation [118]. Furthermore, immunopeptidomic analyses have shown that SLA-DR molecules on infected cells present immunogenic peptides derived from multiple PRRSV-2 nonstructural proteins, and these peptides are recognized by sera from PRRSV-infected piglets [128]. Collectively, these findings indicate that both PRRSV-1 and PRRSV-2 can alter SLA-II-associated antigen presentation, although the molecular mechanisms and immunological consequences require further investigation across different strains and species. Rather than uniformly suppressing antigen presentation, the strains examined may differentially modulate SLA-I and SLA-II pathways, potentially contributing to the delayed and incomplete adaptive immune responses observed during PRRSV infection [127,128].

#### 3.4.2. Immune Checkpoints and T Cell Dysfunction

The prolonged course of PRRSV infection suggests that virus-specific T cells may become functionally restrained [8]. One plausible mechanism is the activation of immune checkpoint pathways and the development of a T cell dysfunction or exhaustion-like state [129]. Immune checkpoint molecules, such as PD-1, CTLA-4, LAG-3, and TIM-3 and their ligands, are critical for maintaining immune homeostasis under physiological conditions, but they can also be exploited during chronic infection to suppress protective immunity [130]. T cell exhaustion is characterized by diminished effector function, sustained expression of inhibitory receptors, impaired recall responses, and a transcriptional profile distinct from that of functional effector or memory T cells [131].

In both PRRSV-1 and PRRSV-2 infection, up-regulation of immune checkpoint molecules has been consistently reported in multiple tissues and across different viral genotypes [132,133,134,135,136]. Increased expression has been documented in the thymus and lung during PRRSV-1 infection, as well as in inguinal lymph nodes, porcine alveolar macrophages, and bronchoalveolar lavage cells during PRRSV-2 infection [122,123,124,125,126]. These observations suggest that checkpoint-mediated immunoregulation is a common feature of PRRSV-induced immune modulation [122,123,124,125,126]. Although many of these data derive from transcriptomic data, functional evidence is beginning to emerge. For example, increased expression of LAG3 has been demonstrated in multiple tissues of PRRSV-2-infected pigs, and modulation of LAG3 signaling in co-culture systems influences PRRSV replication [137]. Collectively, these findings support the idea that immune checkpoint pathways may contribute to T cell dysfunction and viral persistence during PRRSV infection.

## 4. Vaccines Against PRRSV

Vaccination remains one of the most cost-effective strategies for controlling infectious diseases in livestock by reducing disease burden, pathogen transmission, and production losses [17,138]. Commercially available PRRSV vaccines primarily include modified-live virus (MLV) and killed-virus (KV) vaccines, with MLV vaccines being more widely used because they generally induce stronger and more consistent protective immunity [139]. In addition, alternative vaccine platforms, including subunit, mRNA, and viral-vectored vaccines, have been explored to improve the safety and breadth of protection against PRRSV [140,141,142]. Although some subunit vaccines have reached commercial use in specific markets [143], most of these next-generation platforms remain at the experimental or pre-commercial stage.

At a 2007 PRRS vaccine colloquium at the University of Illinois, four key requirements for next-generation vaccines were proposed: rapid induction of immunity, broad protection against circulating PRRSV strains, a favorable safety profile, and the ability to differentiate infected from vaccinated animals (DIVA) [144]. Achieving these goals remains challenging. The extensive genetic and antigenic diversity of PRRSV limits the breadth and predictability of protection against heterologous strains, while the mechanisms underlying heterologous and cross-protective immunity remain incompletely understood [145,146,147,148]. In addition, the viral antigens and epitopes responsible for protective immunity have not been fully defined, constraining the rational design of subunit, mRNA, and viral-vectored vaccines [62,149]. Therefore, future PRRSV vaccine development should focus on identifying conserved protective antigens and immune correlates that can support safe, broadly protective, and DIVA-compatible vaccine platforms [150].

### 4.1. Modified-Live Virus

The first commercial PRRSV modified-live virus (MLV) vaccine, RespPRRS^®^, was introduced in the United States in 1994 [16,151]. Since then, PRRSV MLV vaccines have been widely used. PRRSV-1 MLV vaccines are predominantly used in regions where PRRSV-1 is endemic, particularly in Europe, and some parts of Asia, whereas PRRSV-2 MLV vaccines are mainly used in North America and Asian regions where PRRSV-2 is the predominant circulating species [152]. Most conventional commercial PRRSV MLV vaccines are derived from virulent parental strains through serial passage in permissive cell cultures, particularly MARC-145 cells, resulting in progressive attenuation [151,153]. A major advantage of MLV vaccines is their ability to partially mimic natural PRRSV infection [153]. For both PRRSV-1 and PRRSV-2 MLV vaccines, the attenuated vaccine virus undergoes limited replication in vivo, providing prolonged antigenic stimulation and inducing both humoral and cell-mediated immune responses [17,154]. Both PRRSV-1 and PRRSV-2 MLV vaccines induce virus-specific humoral immune responses, characterized by relatively early seroconversion but delayed and often low-titer neutralizing antibody responses [154,155,156]. In parallel with these humoral responses, both PRRSV-1 and PRRSV-2 MLV vaccines induce virus-specific cellular immune responses involving CD4^+^and CD8^+^ T cells, IFN-γ-producing cells, and, in some studies, γδ T cells, although the magnitude, kinetics, and cross-reactivity of these responses vary among vaccine and challenge strains [154,155,157].

Abundant experimental and field evidence indicates that PRRSV MLV vaccines generally provide the most effective protection among currently available commercial vaccines [153,158,159,160,161,162]. At the individual level, MLVs significantly alleviate fever and respiratory signs, reduce gross and histological lung lesions, and decrease the magnitude and/or duration of viremia [138,163]. In breeding herds, appropriate MLV vaccination can improve sow reproductive performance, including reductions in stillbirths and increases in the number of live-born or weaned piglets [164]. At the population level, by shortening virus-shedding duration and lowering shedding magnitude, MLVs can substantially decrease effective virus transmission within swine herds [139,165].

Protective immunity against PRRSV is multifactorial and remains difficult to predict, particularly given the extensive genetic and antigenic diversity of the virus [138]. Vaccine-induced protection is influenced by many factors such as the specific vaccine–challenge strain combination, host immune background, immunization regimen, and evaluation endpoints [148,154,166,167,168,169]. Overall, both PRRSV-1 and PRRSV-2 commercial modified live virus vaccines can reduce viremia, clinical disease, and viral shedding, thereby alleviating reproductive and respiratory disease [153,157]. However, both within-species heterologous protection and cross-species protection between PRRSV-1 and PRRSV-2 remain variable across vaccine–challenge combinations and experimental settings [161,162,167,169,170].

Based on the refined ORF5 classification, commercially available MLV vaccines represent a limited subset of the genetic diversity of circulating PRRSV strains. PRRSV-1 MLV vaccines are derived exclusively from lineage 1 viruses but represent several sublineages, including L1.1, L1.2, L1.3, and L1.11 [6]. PRRSV-2 MLV vaccines originate from multiple lineages, including L1, L5, L7, and L8 [7]. ORF5-based lineage characterization of circulating viruses can therefore provide valuable information regarding the genetic relationships between field and vaccine strains and may assist in epidemiological surveillance and vaccine evaluation.

Nevertheless, lineage matching alone does not ensure effective protection, particularly for PRRSV-2, which undergoes rapid evolution and continuous diversification, resulting in the emergence and circulation of genetically distinct lineages worldwide [7,171]. Indeed, genetic similarity does not reliably predict cross-protection [172]. For example, protection conferred by an L1D.2-based vaccine against different PRRSV-2 strains did not consistently correlate with ORF5 sequence identity, indicating that genetic distance of ORF5 cannot be directly equated with immunological distance [153]. This is consistent with the fact that protective immunity is not determined by GP5 alone [172], but involves multiple viral antigens and coordinated humoral and cellular responses [173]. Neutralizing antibodies contribute to protection but are often delayed and strain-restricted, whereas T cell responses may exhibit broader cross-reactivity but also vary among strains and hosts [147,174]. Therefore, cross-protection should be evaluated using integrated virological and immunological parameters rather than sequence similarity alone [148,153]. Future vaccine development should prioritize conserved protective epitopes, cross-reactive cellular immunity, mucosal responses, and rational immunogen design to achieve broader and more durable protection against genetically distinct lineages.

Both PRRSV-1 and PRRSV-2 MLV vaccines share common safety concerns, including replication and shedding of vaccine viruses, potential transmission to susceptible pigs, reversion to increased virulence, and recombination with circulating field strains or other MLV strains, which may contribute to the emergence of vaccine-derived variants. The ability of MLV vaccines to replicate in the host is essential for their strong immunogenicity and protective efficacy. However, vaccine virus replication may be accompanied by transient viremia and shedding, creating opportunities for unintended transmission when vaccination and biosecurity practices are not appropriately managed [175,176,177,178]. Moreover, the rapid evolution and high recombination potential of PRRSV provide opportunities for vaccine-derived viruses to exchange genetic materials with other vaccine strains or circulating field viruses, potentially generating variants with altered replication capacity, transmissibility, antigenic properties, or pathogenicity [179,180,181,182,183,184]. Similar to other nidoviruses, PRRSV recombination is mediated by a copy-choice mechanism during discontinuous RNA synthesis, which may occur when multiple strains co-infect the same host [185]. Field studies have confirmed the emergence of recombinant PRRSV-1 and PRRSV-2 variants derived from combinations of MLV strains and/or circulating field strains, particularly in the same herd where different viral lineages coexist [182,183,184]. Although the impact of these recombination events remains uncertain, recombinant viruses with better adaptation, immune escape, or transmission ability may persist in the field and drive PRRSV evolution, raising the risk of reversion to virulence [179,180,181]. These limitations highlight the need for safer and more precisely engineered MLV platforms that retain robust immunogenicity while minimizing the risks associated with uncontrolled replication, genetic instability, and recombination. Reverse genetics has become an important tool for the development of next-generation PRRSV MLVs, enabling rational attenuation, broader cross-protection, immune modulation, and the construction of DIVA-compatible vaccine strains [186,187].

Although MLV vaccines have been used for decades, the molecular mechanisms underlying their attenuation is not fully understood [163]. Conventional PRRSV MLVs, including both PRRSV-1- and PRRSV-2-derived vaccines, are generally generated by serial passage of field isolates in MARC-145 cells or other permissive cell lines [175]. During this process, multiple random mutations and deletions accumulate throughout the viral genome, gradually reducing viral virulence. Although comparative analyses of attenuated PRRSV-2 strains and their parental viruses have identified numerous mutations associated with attenuation, no single conserved genetic determinant has been consistently identified across different vaccine strains [188,189,190]. These results suggest that PRRSV attenuation is a complex multigenic phenotype resulting from the combined effects of multiple genetic changes rather than a single mutation [188,189,190,191]. To overcome the unpredictability associated with conventional attenuation strategies, reverse-genetics systems based on infectious cDNA clones allow rational manipulation of the PRRSV genome through site-directed mutagenesis, sequence deletion or replacement, and modification of transcription-regulatory sequences [187]. These approaches provide a more controlled alternative to attenuation by serial passage. For example, rewiring transcription-regulatory sequences has recently been shown to restrict PRRSV-2 recombination while retaining protective efficacy, providing a potential strategy for developing genetically safer live-attenuated vaccines [192].

Codon-pair deoptimization (CPD) is another promising approach for rational attenuation [193,194]. CPD introduces unfavorable synonymous codon pairs without altering the encoded amino acid sequence, thereby reducing viral protein expression and replication while largely preserving antigenic properties [194]. Gene deoptimization strategies have been explored in both PRRSV-1 and PRRSV-2, targeting viral genes such as GP5, nsp1, nsp9, and ORF7, and have been shown to attenuate viral replication and pathogenicity, and reduce viremia and lung lesions, while retaining sufficient immunogenicity to confer protective immunity [195,196,197,198,199]. Because attenuation is achieved through numerous distributed synonymous substitutions, CPD may also increase the genetic barrier to reversion compared with attenuation determined by only a few mutations [186]. A clearer understanding of the molecular determinants underlying PRRSV attenuation will therefore be essential for the rapid and rational development of vaccines against newly emerging strains. Identifying key attenuation mechanisms may also facilitate the design of genetically stable vaccine strains with a lower risk of reversion to virulence and improved safety profiles [175].

Given the high mutation rate and extensive genetic diversity of PRRSV, vaccines derived from a single strain can hardly provide uniformly effective protection against the diversity of circulating viruses [146,200]. To broaden the spectrum of protection, chimeric vaccine strategies have been developed to combine protective antigenic determinants from genetically distinct strains within a single viral backbone. Using reverse genetics, researchers have constructed chimeric or consensus-based viruses that incorporate immunologically relevant regions from multiple PRRSV strains [201,202]. For example, an attenuated PRRSV-2 expressing a computationally designed consensus sequence spanning ORF2-6 from representative Chinese isolates induced heterologous neutralizing antibodies and provided greater protection against a virulent NADC30-like strain than a conventional modified-live virus (MLV) vaccine [203]. A PRRSV-1/PRRSV-2 chimeric virus was successfully rescued and induced neutralizing antibodies against viruses from both species, providing proof of concept for cross-species antigenic engineering [204]. Similarly, a PRRSV-2 chimera containing NSP2, ORF5, ORF6, and ORF7 sequences from an NADC30-like strain within an HP-PRRSV vaccine backbone conferred cross-protection against both parental antigenic groups in pigs [205]. Together, these findings support the development of rationally designed vaccines that incorporate consensus antigens, conserved protective epitopes, or selected antigenic modules from diverse PRRSV strains, rather than relying solely on a single naturally occurring isolate [96,190,191,192].

DIVA (Differentiating Infected from Vaccinated Animals) vaccines enable serological discrimination between naturally infected and vaccinated animals through defined antigenic markers [206]. Such markers can be introduced by inserting a heterologous antigen (positive marker) or deleting/modifying an immunodominant viral antigen (negative marker) [207]. An ideal DIVA marker should be conserved and immunodominant during natural infection while being dispensable for vaccine viability and protective immunogenicity [208]. Early epitope-mapping studies therefore focused on nsp2, which contains immunodominant regions that can be modified without markedly impairing viral replication [209]. Deletion of nsp2 epitopes enabled serological differentiation between vaccinated and infected animals, although early constructs retained substantial virulence [208]. Subsequent strategies incorporated dual markers and attenuated backbones, but the high sequence variability of nsp2 raised concerns about lineage-dependent diagnostic sensitivity and prompted the search for more conserved markers [210,211]. More recently, replacement of the PRRSV-2 N protein with the antigenically distinct PRRSV-1 N protein, combined with a PRRSV-2 N-specific competitive ELISA, established an integrated DIVA system that also reduced viraemia, lung lesions, and clinical disease after challenge [212]. However, marker conservation, heterologous protection, and the biosafety of modified-live vaccines remain major challenges for PRRSV DIVA strategies. Future strategies should therefore combine reliable serological discrimination with broad antigenic coverage and rational attenuation.

Beyond vaccine strain selection, the effectiveness of PRRSV MLV vaccination can be further improved by optimizing vaccination routes, timing, adjuvant formulation, and herd management [213,214,215,216]. Recent studies suggest that vaccine dose should be optimized rather than simply maximized. Reduced doses of commercial PRRSV-2 MLV vaccines showed comparable or, for some outcomes, improved protection against heterologous NADC30-like and NADC34-like strains [215,216]. However, these effects were strain- and context-dependent. Consistent with this complexity, a recent field-based causal analysis comparing full- and half-dose PRRSV-2 MLV vaccination found production-stage-dependent effects, with full-dose vaccination associated with lower mortality during the finishing phase but not during the nursery phase [217].

Vaccination timing is similarly important. Maternally derived antibodies can interfere with vaccine replication and subsequent adaptive immune responses. Notably, a study showed that a substantial proportion of three-week-old piglets carrying PRRSV-1-specific maternal antibodies failed to mount a detectable antibody response after MLV vaccination, which was associated with reduced protection following challenge [218]. In breeding herds, regimen modification may also enhance passive protection [219].

Route optimization is particularly relevant for improving immune quality and field applicability. Intramuscular administration remains the most widely used route, whereas needle-free intradermal vaccination has shown comparable protective efficacy and may induce earlier cell-mediated responses while providing practical and animal-welfare advantages [220]. Intranasal administration has also been experimentally explored as a means of stimulating mucosal immunity, although its additional benefit over conventional routes are not consistently demonstrated [221,222,223]. In parallel, adjuvant strategies are shifting from conventional immunostimulants toward more targeted approaches, including saponin-based formulations, biomaterial delivery systems, and cytokine-based immunomodulation designed to strengthen early innate and cellular responses and improve heterologous protection [224,225,226,227].

At the herd level, vaccination should therefore be integrated with coordinated gilt acclimatization, breeding-herd vaccination, piglet vaccination, herd closure when appropriate, and strict internal biosecurity [228,229]. Batch-management systems, including three- or five-week batch production, can further support PRRSV control by improving age-group separation, facilitating all-in/all-out production, and allowing more effective cleaning and sanitary downtime between batches [230,231]. Such integrated vaccination and management programs have been successfully incorporated into PRRSV stabilization strategies in commercial farms [232].

Collectively, these findings suggest that future PRRSV vaccination strategies should integrate antigen design with coordinated optimization of dose, schedule, route, and adjuvant formulation. An ideal regimen should minimize maternal-antibody interference, induce rapid systemic and mucosal immunity, promote durable cellular responses, and broaden protection against genetically diverse field strains while maintaining safety, practicality, and production efficiency [14].

### 4.2. Inactivated Vaccines

Commercial inactivated vaccines are also available for PRRSV and offer a clear safety advantage over MLVs, particularly in pregnant sows and herds with stringent biosecurity requirements [14]. However, their major limitation is weak immunogenicity [14]. In most settings, inactivated vaccines induce detectable PRRSV-specific antibody responses but elicit only limited neutralizing antibody production and relatively poor cellular immunity, especially in the absence of potent adjuvants or repeated boosting [233,234].

This limited efficacy likely reflects not only the inherently lower immunostimulatory capacity of non-replicating antigens, but also the structural fragility of PRRSV envelope proteins [235]. Conformational epitopes relevant to neutralization may be altered or lost during virus inactivation, thereby reducing the ability of the vaccine to reproduce the antigenic features required for robust protective immunity. As a result, inactivated vaccines often provide only modest effects on viremia, viral shedding or heterologous challenge outcome [236].

Nevertheless, these vaccines may still have value in specific contexts. In previously primed animals, especially breeding females with prior exposure to PRRSV-1 antigens, inactivated vaccines can function as boosters and help reinforce herd immunity [237]. Their utility may therefore lie less in primary immunization and more in maintaining immune stability, improving reproductive performance or reducing the risk associated with live vaccine administration [219,238]. Their strong safety profile also makes them attractive candidates for formulation improvement through optimized adjuvants, nanoparticle delivery systems and structurally informed antigen design [239,240,241].

### 4.3. Next-Generation Vaccine Platforms

#### 4.3.1. Subunit, DNA and Viral-Vectored Vaccines

Protein subunit vaccines have emerged as a major focus of next-generation PRRSV vaccine development because, compared with MLVs, they offer several conceptual advantages, including improved safety, compatibility with DIVA strategies and greater flexibility for rational antigen design [15]. Recombinant antigens derived from both PRRSV-1 and PRRSV-2 have been produced in diverse expression systems, including bacterial, yeast, insect, mammalian and plant-based platforms [242,243,244,245,246,247,248]. Studies using baculovirus-expressed proteins and plant-derived oral formulations have demonstrated that both PRRSV-1 and PRRSV-2 subunit vaccines can induce measurable humoral responses and, to some extent, cellular immunity [249,250]. Although PRRSV subunit vaccines offer excellent safety profiles, their application remains limited by insufficient immunogenicity, weak induction of neutralizing antibodies, uncertainty regarding protective antigen selection, limited cross-protection against genetically diverse PRRSV strains, and the requirement for potent adjuvants or advanced delivery platforms [149,236]. This problem reflects not only the limited immunogenicity of isolated proteins, but also the challenge of reproducing the structural and antigenic complexity of the native virion [11,251]. Accordingly, recent work has moved away from simplified single-antigen formulations towards more sophisticated multicomponent and structurally optimized designs [252].

This limitation has also shaped efforts to develop DNA vaccines and viral-vectored vaccines as alternatives to conventional PRRSV vaccines [252]. These platforms are attractive because they are flexible in manufacture, readily accommodate the incorporation of multiple antigens and are compatible with the use of molecular adjuvants [253,254,255]. However, their protective efficacy remains inconsistent and is highly dependent on the choice, expression level, and native conformation of the inserted antigens [256,257]. Moreover, vector-specific biosafety and regulatory concerns must also be considered [242]. Importantly, although viral-vector and DNA-based vaccine platforms provide flexible antigen delivery strategies, they do not fundamentally overcome the extensive antigenic diversity of PRRSV, and broad heterologous protection remains difficult to achieve and has not been consistently demonstrated [13,242]. A broad range of viral vectors, including adenoviruses, herpesviruses, Newcastle disease virus, vaccinia virus, pseudorabies virus and baculovirus-derived systems, has been explored for both PRRSV-1 and PRRSV-2 antigen delivery [256,257,258,259,260]. In experimental models, these vectored platforms have been able to induce both humoral responses, including neutralizing antibodies, and detectable cellular immunity, underscoring their value as adaptable vaccine scaffolds [261,262]. However, as with subunit vaccines, their protective efficacy depends critically on antigen composition and on how effectively they reproduce the structural and immunological features of authentic viral infection. For DNA vaccines in particular, molecular adjuvants such as IL-2, IL-4, IL-18 and type I or type II interferons have been used to enhance immunogenicity [263,264,265,266,267]. Overall, IL-2, IL-18 and IFN-α/γ generally promote stronger cellular immune responses, whereas IL-4 appears to exert more context-dependent effects, sometimes benefiting humoral responses but impairing cell-mediated immunity [263,264,265,266,267].

Taken together, although DNA vaccine and viral-vectored vaccines offer considerable advantages in safety and antigen-design flexibility, further optimization of protective antigen combinations, vector platforms, and vaccination regimens, together with large-scale field validation, is required before their widespread application in commercial swine production.

#### 4.3.2. Antigen Design

For subunit, DNA, and viral vectored vaccines alike, antigen selection represents a critical determinant of vaccine efficacy. Early next-generation PRRSV vaccine strategies focused predominantly on GP5, which was long regarded as the principal target of neutralizing antibodies [45]. However, it is now clear that GP5 alone is insufficient to reproduce the conformational and multicomponent antigenic architecture of the native viral envelope [268,269,270]. This realization has prompted a shift away from single-antigen constructs towards multicomponent formulations. Co-expression of GP5 and M enhances immunogenicity relative to GP5 alone, and the GP5/M heterodimer is now widely considered a central antigenic unit in PRRSV vaccine development [47]. Likewise, the incorporation of minor envelope glycoproteins, particularly GP3 and GP4, can further improve immune responses [252]. For example, recombinant adenoviral vaccines expressing PRRSV-2 GP3-GP5 or GP3-GP4-GP5 fusion constructs induce stronger neutralizing antibody responses, lymphocyte proliferation and cytotoxic T lymphocyte activity than their single-antigen counterparts [252]. These findings collectively support the view that broader representation of envelope antigens is likely to be essential for improved protection.

A major challenge common to both subunit and vectored vaccines is the reconstruction of antigenic structures that faithfully resemble those of the native virion [15]. In this regard, a recent vector vaccine based on a Venezuelan equine encephalitis virus replicon pseudotyped with vesicular stomatitis virus glycoprotein represents an important technical advance [271]. This platform encoded GP3, GP4, GP5 and M of the NADC30-like PRRSV GXGG-8011 strain, enabled stable antigen expression and promoted the formation of virus-like vesicles in vitro, thereby improving structural mimicry of the native envelope [271]. In piglets, intramuscular immunization induced strong humoral responses comparable to those elicited by inactivated vaccines, together with a predominantly Th2-skewed cellular profile, attenuated induction of inflammatory cytokines, reduced viremia and markedly alleviated lung pathology after challenge [271]. Because these virus-like vesicles are non-integrating and non-replicative, this approach may offer a more favourable safety profile than live replicating vectors while preserving structural features that are likely to be relevant to protective immunity.

An increasing number of studies have highlighted the importance of cell-mediated immune responses in protection conferred by PRRSV vaccines [13]. Accordingly, vaccine research has increasingly focused on improving the presentation of T cell epitopes [271,272]. A self-assembling ferritin nanoparticle vaccine displaying GP3-, GP4- and GP5-derived epitopes from the NADC30-like strain FJ1402 has been shown to activate both CD4^+^ and CD8^+^ T cells, induce antigen-specific IFN-γ secretion and reduce viral loads, lung injury and clinical signs in piglets challenged with PRRSV [273]. Similarly, a multicomponent Fc-fused subunit vaccine composed of GP4Fc, GP5Fc and MFc, produced in CHO cells, elicited robust humoral and cellular immune responses and conferred cross-protection against both QYYZ-like and NADC30-like strains [272]. These studies illustrate an important conceptual advance in the field: rather than relying on a single dominant antigen, next-generation subunit vaccines are increasingly being designed to better mimic the antigenic complexity of the virion while simultaneously improving antigen stability, immunogenicity and breadth.

#### 4.3.3. mRNA Vaccines

mRNA vaccines have rapidly emerged as a powerful vaccine platform and are increasingly viewed as a promising strategy for the prevention and control of PRRS [273,274]. By delivering antigen-encoding RNA into host cells, mRNA vaccines enable in situ antigen expression and presentation, thereby eliciting both humoral and cellular immune responses [275]. Their principal advantages include rapid and adaptable antigen design, platform-based and scalable manufacturing, a favorable safety profile, and the capacity to encode multivalent or structurally engineered immunogens [276,277].These properties are especially attractive for PRRSV, given its extensive genetic diversity, rapid evolution, and capacity for immune evasion [278].

mRNA vaccines can be broadly divided into non-amplifying mRNA and self-amplifying RNA (saRNA) platforms [279]. Whereas non-amplifying mRNA resembles endogenous mature mRNA in structure, saRNA additionally encodes replicase machinery that enables intracellular RNA amplification and prolonged antigen expression at lower doses [280]. In both cases, however, the intrinsic instability of naked RNA necessitates the use of efficient delivery systems [281]. Among currently available platforms, lipid nanoparticles (LNPs) have emerged as the dominant vehicle owing to their ability to protect RNA from degradation, promote cellular uptake, and facilitate cytoplasmic release, while remaining compatible with large-scale production [282].

Recent studies have begun to provide proof of concept for mRNA-based PRRSV-2 vaccines [278,283,284,285]. In contrast, reports on mRNA-based PRRSV-1 vaccines remain limited. Lipid nanoparticle-formulated mRNA vaccines encoding PRRSV structural proteins can induce protective immunity in piglets, and multicomponent formulations, especially those combining GP5, M, and N, appear to outperform fusion-antigen constructs in both humoral and cellular immunogenicity [283]. A multiepitope mRNA vaccine was designed by combining immunoinformatics predictions and literature-reported B cell and T cell epitopes from HP-PRRSV structural proteins [284]. Compared with an inactivated vaccine, the mRNA vaccine improved piglet survival, and reduced viremia and lung lesions, providing new insights for future PRRS vaccine development [284]. More recently, an mRNA-LNP vaccine targeting NADC30-like PRRSV identified a GP345m-based fusion construct as the optimal immunogen [278]. Notably, the authors reported that vaccine conferred substantial protection in piglets and induced PRRSV-specific cellular immunity despite the absence of detectable neutralizing antibodies [278]. This observation is particularly important because it suggests that, in the context of genetically diverse PRRSV strains, robust cellular immunity may serve as a key correlate of protection even when classical neutralizing antibody responses are weak or delayed.

Collectively, the major advantages of this platform include the absence of infectious vaccine virus, rapid and modular antigen design, and compatibility with multivalent formulations [273,286]. However, several challenges remain before mRNA-based vaccines can be translated into practical applications for PRRSV control [15]. Optimal antigen selection and combination strategies, durability and consistency of protection, and breadth of heterologous immunity against genetically diverse PRRSV strains remain insufficiently characterized [14,274]. Furthermore, large-scale field validation, cost-effective manufacturing, storage stability, and feasible delivery strategies under commercial swine production conditions require further investigation [11,283,284]. Therefore, although mRNA vaccines represent an attractive strategy for overcoming some of the safety and antigenic-matching limitations of conventional PRRSV vaccines, further optimization and large-scale validation are required before their widespread application in the field [285].

PRRSV vaccine development remains challenging because of the virus’s high genetic and antigenic diversity and its ability to evade host immunity, which limit the cross-protection provided by conventional vaccines [14,114]. A summary of the major PRRSV vaccine platforms and their respective advantages and limitations is provided in Table 1. Recent efforts are increasingly focusing on more rational antigen design, supported by immunoinformatics, structural analysis, and a better understanding of PRRSV-host interactions [150]. In this context, multiepitope strategies and mRNA vaccine platforms may provide useful directions for developing safer, broader, and more durable PRRSV vaccines [11,14,242].

## 5. Antiviral Strategies Against PRRSV

### 5.1. Direct-Acting Antivirals Targeting Viral Proteins

**Figure 5:**
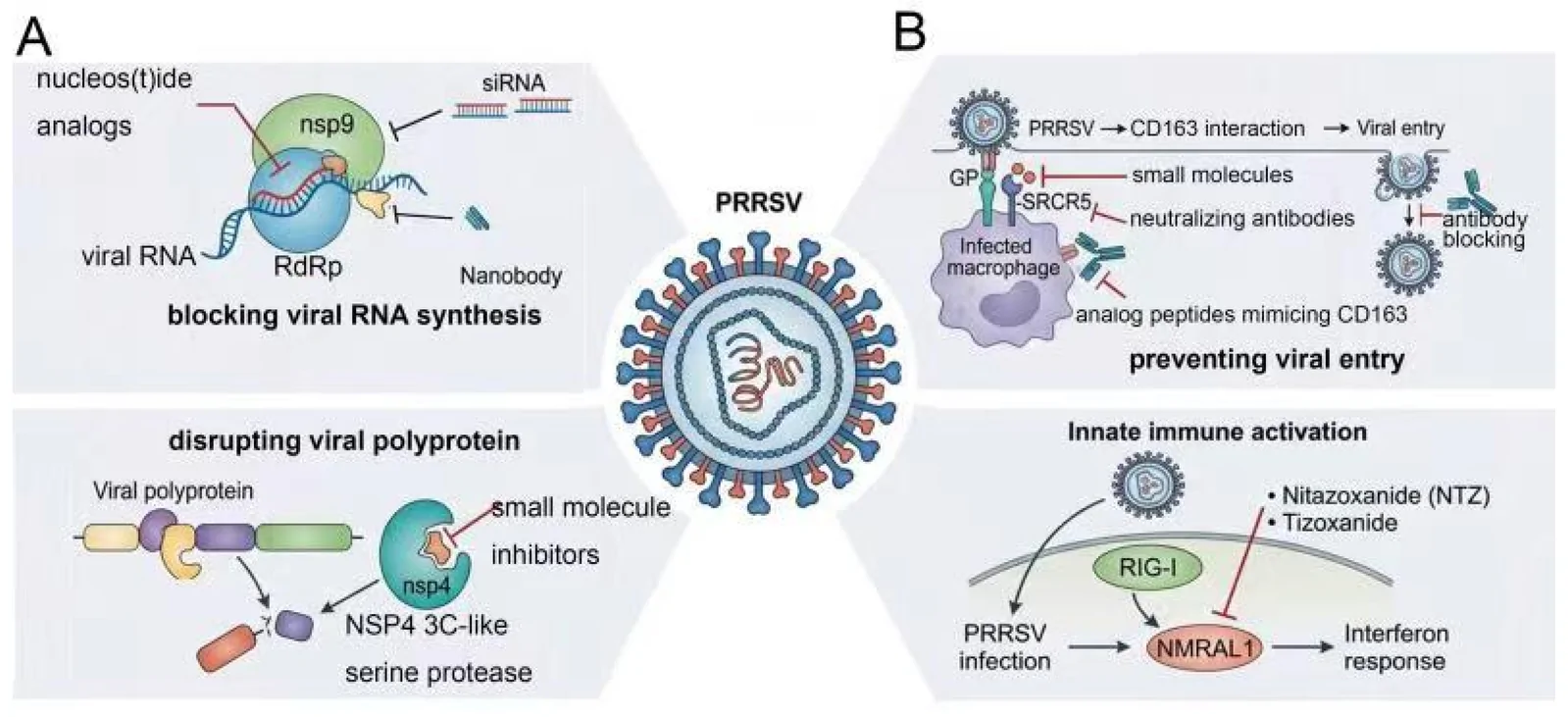
Emerging antiviral molecule development strategies for PRRSV control: targeting viral machinery, host dependency factors, and genetic resistance. (**A**) Viral-targeted antiviral approaches. Direct-acting antivirals target essential PRRSV replication components. Inhibition of the nsp9 RNA-dependent RNA polymerase (RdRp) is achieved through nucleos(t)ide analogs (remdesivir, GS-441524, molnupiravir, and ribavirin), siRNA-mediated suppression, and nanobody-based blockade, resulting in impaired viral RNA synthesis. Targeting the nsp4 3C-like serine protease with small-molecule inhibitors, including flavonol derivatives, disrupts viral polyprotein processing and replication complex formation. (**B**) Host factor-directed antiviral strategies. Host-targeted interventions block critical cellular processes required for PRRSV infection. CD163-mediated viral entry can be inhibited through SRCR5-targeting molecules, neutralizing antibodies, and CD163-derived peptides. In addition, innate immune modulation by nitazoxanide (NTZ) and tizoxanide enhances RIG-I–mediated interferon signaling through NMRAL1 targeting, restoring antiviral host responses. Collectively, these strategies represent next-generation PRRSV control approaches by targeting viral replication machinery, host dependency factors, and immune pathways, offering alternatives to conventional vaccine-based prevention. This figure was created in BioRender https://BioRender.com/dmjwdtb. Agreement number BF2A90XC18.

Because current vaccines do not completely block PRRSV transmission, increasing attention has been directed toward the development of antiviral therapeutics as a complementary strategy to vaccination [60]. Although this topic has been reviewed elsewhere, here we briefly summarize recent progress in the development of anti-PRRSV strategies [1,287,288,289]. In general, these antiviral strategies can be divided into two broad categories: those that directly target viral proteins or the viral genome, and those that target host factors required for viral infection or replication (Figure 5).

Among viral targets, nonstructural protein 9 (nsp9), the viral RNA-dependent RNA polymerase (RdRp), has attracted considerable interest because of its central role in genome replication and transcription [23]. Pharmacological inhibition of nsp9 is therefore expected to directly impair viral RNA synthesis [290]. Using a GFP-expressing PRRSV reporter virus assay, one study demonstrated that several RdRp-targeting nucleos(t)ide analogs, including remdesivir, GS-441524, molnupiravir, and ribavirin, exert marked inhibitory effects against both PRRSV-1 and PRRSV-2 [290]. Structural comparison of PRRSV nsp9 with SARS-CoV-2 nsp12 further suggests that conservation within the catalytic core of RdRp may provide a mechanistic explanation for the cross-viral activity of some polymerase inhibitors [290]. Beyond small molecules, gene-silencing approaches have also validated nsp9 as a tractable target, as multiple siRNAs directed against nsp9 substantially reduced viral replication and nsp9 expression in MARC-145 cells [291].

Natural products have also been explored as nsp9-targeting antivirals [292]. An aqueous extract from the fruiting bodies of Cryptoporus volvatus was reported to inhibit multiple PRRSV-2 strains in both MARC-145 cells and porcine alveolar macrophages (PAMs) in a dose-dependent manner, with low cytotoxicity and a favorable therapeutic window [292]. Mechanistic analyses suggested that this extract directly inhibits RdRp activity of nsp9, consistent with reduced viral RNA synthesis and protein expression in infected cells. Importantly, in piglets challenged with a highly pathogenic PRRSV strain, treatment alleviated clinical symptoms, reduced serum viremia, and improved survival [292]. These highlight the in vivo potential of volvatus-derived products as antiviral candidates.

Biologics targeting nsp9 further support this viral polymerase as a promising antiviral target [293,294,295]. In particular, an nsp9-specific nanobody showed inhibitory activity against several PRRSV-2 strains, and subsequent engineering strategies improved its intracellular delivery and tropism for PRRSV-susceptible macrophages [293]. The optimized construct suppressed viral infection in porcine alveolar macrophages through direct targeting of nsp9 and may also exert immunomodulatory effects [294,295]. Collectively, these findings suggest that nsp9-targeting biologics represent a potentially useful complement to small-molecule antiviral approaches.

Another direct-acting antiviral target is nsp4, the viral 3C-like serine protease that processes the replicase polyprotein and is indispensable for formation of the replication-transcription complex [21]. Inhibiting nsp4 therefore disrupts an early and essential step in the viral life cycle [16]. Early studies combining enzymatic screening and cell-based assays identified small molecules that inhibited nsp4 protease activity and reduced HP-PRRSV replication in MARC-145 cells, providing proof of concept for this strategy [296]. More recently, researchers optimized a class of natural-like compounds called flavonol derivatives and identified one candidate-compound 4s, which potently inhibits classic PRRSV CH1a strain while showing low toxicity [297]. Experimental evidence together with molecular modeling suggested that its antiviral activity is mediated by binding to and suppressing the viral nsp4 protease [298]. In parallel, structure-guided virtual screening based on available nsp4 structural information has proposed additional candidate scaffolds [299]. However, most of these in silico hits still require biochemical validation, confirmation of cellular target engagement, and antiviral testing in relevant experimental systems [300].

### 5.2. Host-Directed Antiviral Strategies

Host-directed antiviral strategies are also being actively studied, particularly those targeting viral entry factors or innate immune regulators [1]. CD163 is essential for PRRSV infection and has therefore become a major target for generating PRRS-resistant pigs [301]. It was initially demonstrated that CRISPR/Cas9-mediated disruption of CD163 conferred resistance to PRRSV, and subsequent studies confirmed that complete CD163 knockout can provide complete resistance to highly pathogenic PRRSV strains [301]. However, because CD163 is involved in important physiological processes, including hemoglobin-haptoglobin clearance and immune regulation, research has increasingly shifted toward precise editing strategies that block viral entry while preserving receptor function [302]. The fifth scavenger receptor cysteine-rich domain (SRCR5), encoded by exon 7, was identified as a critical determinant of PRRSV susceptibility [303]. Deletion of SRCR5 or exon 7 confers resistance to PRRSV-1 and PRRSV-2 while largely maintaining normal CD163 function [281,304,305]. SRCR5-deficient pigs also showed normal growth, health, reproductive performance, and meat composition [306]. More recently, deletion of exon 13 encoding the PSTII domain resulted in complete resistance to PRRSV-2 infection in vivo, and PAMs derived from ΔExon13 pigs were fully resistant to both PRRSV-1 and PRRSV-2 infections in vitro [307]. The E529G substitution markedly reduced HP-PRRSV replication while retaining major CD163 functions [308]. Simultaneous editing of CD163 and ANPEP has also generated pigs resistant to both PRRSV-2 and transmissible gastroenteritis virus, demonstrating the potential of multiplex genome editing for broad-spectrum disease resistance [309]. CD163-based PRRS resistance has also progressed toward commercial application. In 2025, CD163ΔE7 pigs received FDA approval for commercial use, with subsequent Canadian assessments supporting their safety equivalence to conventional pigs [310]. Similar regulatory progress has also been reported in several Latin American countries. These milestones highlight exon 7/SRCR5 deletion as one of the most advanced genome-editing strategies for commercial PRRS-resistant pig breeding.

Despite these advances, several challenges remain. The effectiveness of different CD163 modifications may vary among PRRSV genotypes and strains, and some modifications are insufficient to confer complete resistance [301,311]. Although recent studies indicate that SRCR5-edited pigs maintain normal growth, reproductive performance, and meat characteristics, further large-scale, multigenerational evaluations under commercial production conditions are necessary [306,312]. Potential unintended genomic alterations should also be carefully assessed using comprehensive genomic analyses [313,314]. From a breeding perspective, the edited allele must be stably introduced into elite commercial populations without compromising economically important traits, while maintaining genetic diversity and production efficiency [312,315]. In addition, differences in regulatory frameworks, international trade policies, and public acceptance may influence the global adoption of genome-edited livestock [316,317,318]. Therefore, future studies should emphasize durable resistance, long-term safety, genetic stability, production performance, and field-level effectiveness to support the broader application of CD163-edited pigs (Figure 5).

Small molecules that directly bind to CD163-SRCR5 and disrupt its interaction with viral glycoproteins have shown antiviral activity against both genotype 1 and genotype 2 PRRSV strains in vitro [319]. Antibodies and peptides derived from critical CD163 regions have likewise demonstrated inhibitory effects on viral attachment and entry. Broadly neutralizing monoclonal antibodies 8H2 and 4H7 significantly inhibited the attachment of multiple PRRSV-2 strains, reducing viral titers by 100- to 1000-fold and viral RNA levels by approximately 10^4^-fold [320]. These antibodies recognized linear epitopes located within SRCR6 and the PSTI domain, and mutations in these regions abolished antibody recognition. Peptides derived from these epitopes displayed broad, dose-dependent antiviral activity and suppressed PRRSV-associated NF-κB signaling [320,321].

Additional host-directed approaches seek not to block entry directly, but to enhance endogenous antiviral immunity. Our group identified nitazoxanide (NTZ) and its active metabolite tizoxanide as potent inhibitors of PRRSV-2 replication that act by targeting the host factor NMRAL1 and enhancing RIG-I-dependent antiviral signaling. In experimental pig infections, this approach reduced viremia, lung lesions and mortality, illustrating the potential value of host-targeted immunomodulation [322].

Overall, current progress in anti-PRRSV drug development highlights two complementary principles: direct inhibition of essential viral enzymes and modulation of host factors involved in viral entry or innate immunity [288,323]. Although most candidates remain at the preclinical stage, these strategies support the emerging concept that antiviral interventions may serve as complementary approaches to vaccination and biosecurity measures in integrated PRRSV control programs [1,289].

**Figure 6:**
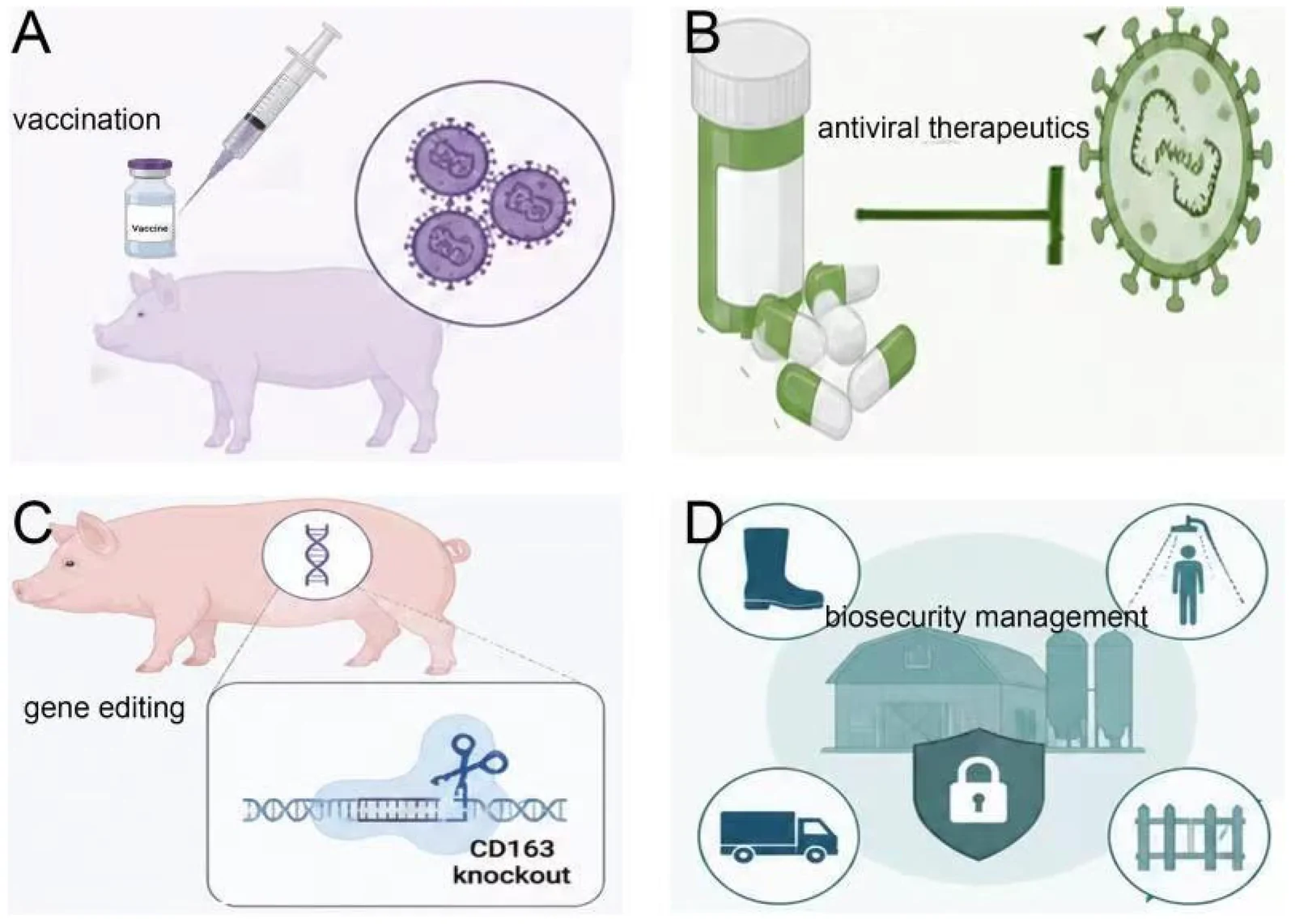
Integrated strategies for PRRSV control: vaccination, antiviral therapeutics, CD163 gene editing, and biosecurity management. An overview of the major approaches currently explored for controlling PRRSV infection. The integrated framework highlights four complementary strategies, including (**A**) vaccination, (**B**) antiviral therapeutics, (**C**) CD163 gene editing pigs, and (**D**) biosecurity management, which collectively contribute to reducing viral transmission, limiting infection, and improving disease control. This figure was created in BioRender https://BioRender.com/u7cv2u6. Agreement number BF2A90YPLV.

Moreover, farm management practices remain essential for PRRSV control, as biosecurity, herd closure, animal-flow management, acclimatization, surveillance, and regional control programs strongly influence the effectiveness of vaccination under field conditions [215,309]. Therefore, future PRRSV control should be considered an integrated strategy combining vaccination, targeted supportive interventions, and rigorous management practices (Figure 6) [215,324,325,326].

## 6. Discussion

The evidence summarized in this review indicates that protection against PRRSV is multifactorial and cannot be explained by a single immune parameter, highlighting the importance of coordinated humoral and cellular immune responses. Neutralizing antibodies are clearly important, but their delayed appearance, restricted breadth and susceptibility to viral escape limit their predictive value when considered in isolation. At the same time, cell-mediated immunity, particularly functionally relevant CD4+ and CD8+ T cell responses, is increasingly recognized as a critical but still incompletely studied contributor to protection. This has important implications for vaccine design, as future platforms will need to improve not only antigenic breadth and structural mimicry, but also the quality and durability of T cell immunity.

This immunological complexity presents a major obstacle to PRRSV vaccine development. Although modified-live virus (MLV) vaccines remain the most effective option for reducing clinical disease, viremia, and virus shedding, their protection against heterologous strains is inconsistent and concerns over genetic instability, recombination, and reversion to virulence persist. Farm-specific strategies, including autogenous vaccines and live-virus immunization, may improve protection against local strains but face limitations in efficacy, biosafety, and regulatory approval. By contrast, non-replicating platforms-including subunit, vectored, nanoparticle, and mRNA vaccines provide improved safety and greater flexibility in antigen design but have yet to achieve the breadth and durability of immunity elicited by live-virus vaccines. Given the extensive genetic and antigenic diversity of PRRSV, sequence similarity alone cannot reliably predict cross-protection. Accordingly, future vaccine evaluation should integrate multiple correlates of protection, including neutralizing breadth, functional T cell responses, mucosal immunity, long-term durability, and protection against genetically diverse challenge strains.

Finally, the findings reviewed here indicate that vaccination alone is unlikely to provide complete control of PRRSV. Emerging antiviral approaches targeting viral replication machinery, host entry factors, or antiviral signaling pathways may complement vaccination by reducing viral replication and transmission, particularly during outbreaks or periods of high infection pressure. Their eventual value, however, will depend on demonstrating efficacy, safety, practicality, and cost-effectiveness under commercial production conditions. Thus, a more realistic framework for PRRSV control is likely to integrate improved vaccines with surveillance, biosecurity, and herd-management measures rather than relying on a single intervention.

## 7. Conclusions

PRRSV remains exceptionally difficult to control because of its extensive genetic diversity, capacity for immune evasion and ability to establish prolonged infection in the face of measurable adaptive immune responses. Evidence reviewed here highlighted that protection against PRRSV is a multifactorial outcome shaped by the coordinated contribution of humoral and cellular immunity, antigen selection, and vaccine platform design. Therefore, no single immune parameter or vaccine strategy can fully predict protective efficacy. Instead, effective control of PRRSV will likely depend on a more integrated understanding of how antigenic variation, immune evasion and protective immunity interact across different viral strains and host contexts.

Considerable progress has been made in understanding the mechanisms of PRRSV adaptive immune dysregulation and in developing improved vaccine and antiviral strategies. Nevertheless, currently available vaccines provide variable heterologous protection, whereas most next-generation vaccine and antiviral approaches remain at the experimental or preclinical stage. A deeper mechanistic understanding of protective immunity and viral immune evasion will be essential for translating emerging technologies into more effective disease-control strategies.

## 8. Future Perspectives

Future progress in PRRSV control will require a better understanding of the immune responses that provide effective protection. In addition, identifying conserved protective epitopes and antigens among different PRRSV strains will be important for the development of vaccines that provide broader and more consistent protection.

From a translational perspective, progress is now occurring along three converging approaches: rational redesign of live vaccines to improve safety and genetic stability, development of next-generation non-replicating platforms with enhanced antigen presentation and epitope design, and exploration of antiviral drugs as complementary tools for reducing viral replication and transmission. The next major challenge will be to connect these advances through mechanistically grounded correlates of protection that are robust across genetically diverse PRRSV strains. Achieving this goal will be essential for moving PRRSV control from partial suppression toward predictable, durable prevention and eventual elimination.

## Figures and Tables

**Table 1:** Overview of major PRRSV vaccine platforms and their characteristics.

Platform	Representative Antigen/Design	Main Immune Response	Main Advantage	Main Limitation	Key References
MLV	attenuated whole virus	strong humoral + cellular	best field efficacy	reversion/recombination risk	[157,158,170,175]
Inactivated	killed whole virus	limited immunity	safety	weak protection	[234,236,238]
Subunit	GP5, GP5/M, GP3/4/5	mainly humoral, variable cellular	safety, DIVA	limited protection	[243,246,251]
DNA	structural proteins + cytokine adjuvants	mainly cellular, variable humoral	flexible design	variable efficacy	[253,254,255]
Viral vector	adenovirus, NDV, etc.	humoral + cellular	multivalent potential	antigen dependence	[257,259,262]
mRNA	GP5/M/N or multiepitope	cellular + humoral	rapid redesign	still early-stage	[279,284,285]

## Data Availability

All the data referred are available upon request to the correspondent author.

## References

[1] Zhang X., Chen Y., Liu M., Long X., Guo C. (2024). Intervention strategies targeting virus and host factors against porcine reproductive and respiratory syndrome virus: A systematic review. Int. J. Biol. Macromol..

[2] Torrents D., Miranda J., Gauger P.C., Ramirez A., Linhares D. (2021). Effect of PRRSV stability on productive parameters in breeding herds of a swine large integrated group in Spain. Porc. Health Manag..

[3] Majumder S., Saha M. (2025). Porcine Reproductive and Respiratory Syndrome (PRRS): A Comprehensive Review of Recent Developments in Diagnosis and Control Strategies. J. Adv. Biol. Biotechnol..

[4] Li H., Lv C., Wang R., Zhao X., Huang L., Zhao M. (2026). Advances in the immunosuppression of porcine reproductive and respiratory syndrome virus. Front. Vet. Sci..

[5] Shi M., Lam T.T.Y., Hon C.C., Hui R.K.H., Faaberg K.S., Wennblom T., Murtaugh M.P., Stadejek T., Leung F.C.C. (2010). Molecular epidemiology of PRRSV: A phylogenetic perspective. Virus Res..

[6] Yim-im W., Anderson T.K., Böhmer J., Baliellas J., Stadejek T., Gauger P.C., Krueger K.M., Vermeulen C.J., Buter R., Kazlouski A. (2025). Refining genetic classification of global porcine reproductive and respiratory syndrome virus type 1 (PRRSV-1) and investigating their geographic and temporal distributions. Vet. Microbiol..

[7] Yim-Im W., Anderson T.K., Paploski I.A.D., VanderWaal K., Gauger P., Krueger K., Shi M., Main R., Zhang J. (2023). Refining PRRSV-2 genetic classification based on global ORF5 sequences and investigation of their geographic distributions and temporal changes. Microbiol. Spectr..

[8] Ma S., Mao Q., Weng S., Lian K., Jiang Z., Hao J., Zhang K. (2026). PRRSV subverts host programmed cell death network for immune evasion. Virulence.

[9] Li Y., Chen W., Wu Q., Tang J., Chen M., Liang H., Liang X., Zhang Y., Liu K. (2026). PRRSV interaction with the adaptive immune system of host: An update review. Virology.

[10] He Z., Yan J., Liu M., Liao J., Li F., Guo C. (2025). Host innate immune antagonism and immune evasion strategies of porcine reproductive and respiratory syndrome virus. Int. J. Biol. Macromol..

[11] Gao F., Wen G. (2025). Strategies and scheming: The war between PRRSV and host cells. Virol. J..

[12] Fiers J., Cay A.B., Maes D., Tignon M. (2024). A Comprehensive Review on Porcine Reproductive and Respiratory Syndrome Virus with Emphasis on Immunity. Vaccines.

[13] He Z., Li F., Liu M., Liao J., Guo C. (2025). Porcine Reproductive and Respiratory Syndrome Virus: Challenges and Advances in Vaccine Development. Vaccines.

[14] Sun Y., Han M., Kim C., Calvert J.G., Yoo D. (2012). Interplay between interferon-mediated innate immunity and porcine reproductive and respiratory syndrome virus. Viruses.

[15] Rahe M.C., Murtaugh M.P. (2017). Mechanisms of Adaptive Immunity to Porcine Reproductive and Respiratory Syndrome Virus. Viruses.

[16] Wang H., Feng W. (2024). Current Status of Porcine Reproductive and Respiratory Syndrome Vaccines. Vaccines.

[17] Li J., Miller L.C., Sang Y. (2024). Current Status of Vaccines for Porcine Reproductive and Respiratory Syndrome: Interferon Response, Immunological Overview, and Future Prospects. Vaccines.

[18] Fang Y., Snijder E.J. (2010). The PRRSV replicase: Exploring the multifunctionality of an intriguing set of nonstructural proteins. Virus Res..

[19] Li Y., Tas A., Sun Z., Snijder E.J., Fang Y. (2015). Proteolytic processing of the porcine reproductive and respiratory syndrome virus replicase. Virus Res..

[20] Tian D., Wei Z., Zevenhoven-Dobbe J.C., Liu R., Tong G., Snijder E.J., Yuan S. (2012). Arterivirus minor envelope proteins are a major determinant of viral tropism in cell culture. J. Virol..

[21] Mardassi H., Massie B., Dea S. (1996). Intracellular synthesis, processing, and transport of proteins encoded by ORFs 5 to 7 of porcine reproductive and respiratory syndrome virus. Virology.

[22] Tian X., Lu G., Gao F., Peng H., Feng Y., Ma G., Bartlam M., Tian K., Yan J., Hilgenfeld R. (2009). Structure and cleavage specificity of the chymotrypsin-like serine protease (3CLSP/nsp4) of Porcine Reproductive and Respiratory Syndrome Virus (PRRSV). J. Mol. Biol..

[23] Liu Y., Hu Y., Chai Y., Liu L., Song J., Zhou S., Su J., Zhou L., Ge X., Guo X. (2018). Identification of Nonstructural Protein 8 as the N-Terminus of the RNA-Dependent RNA Polymerase of Porcine Reproductive and Respiratory Syndrome Virus. Virol. Sin..

[24] Sha H., Zhang H., Chen Y., Huang L., Zhao M., Wang N. (2022). Research Progress on the NSP9 Protein of Porcine Reproductive and Respiratory Syndrome Virus. Front. Vet. Sci..

[25] Nan H., Lan J., Tian M., Dong S., Tian J., Liu L., Xu X., Chen H. (2018). The Network of Interactions Among Porcine Reproductive and Respiratory Syndrome Virus Non-structural Proteins. Front. Microbiol..

[26] Chen Z., Lawson S., Sun Z., Zhou X., Guan X., Christopher-Hennings J., Nelson E.A., Fang Y. (2010). Identification of two auto-cleavage products of nonstructural protein 1 (nsp1) in porcine reproductive and respiratory syndrome virus infected cells: Nsp1 function as interferon antagonist. Virology.

[27] Zheng Y., Jiang D., Sui C., Wu X., Hu Y., Lee C., Cong X., Li J., Lu Y., Wang Z. (2024). PRRSV NSP1α degrades TRIM25 through proteasome system to inhibit host antiviral immune response. Vet. Microbiol..

[28] Li J., Zhang J., Sun P., Wang J., Li G., Cui Z., Li D., Yuan H., Wang T., Li K. (2024). Porcine reproductive and respiratory syndrome virus nonstructural protein 2 promotes the autophagic degradation of adaptor protein SH3KBP1 to antagonize host innate immune responses by enhancing K63-linked polyubiquitination of RIG-I. PLoS Pathog..

[29] Yang L., He J., Wang R., Zhang X., Lin S., Ma Z., Zhang Y. (2019). Nonstructural Protein 11 of Porcine Reproductive and Respiratory Syndrome Virus Induces STAT2 Degradation To Inhibit Interferon Signaling. J. Virol..

[30] Wang D., Chen J., Yu C., Zhu X., Xu S., Fang L., Xiao S. (2019). Porcine Reproductive and Respiratory Syndrome Virus nsp11 Antagonizes Type I Interferon Signaling by Targeting IRF9. J. Virol..

[31] Wissink E.H., Kroese M.V., van Wijk H.A., Rijsewijk F.A., Meulenberg J.J., Rottier P.J. (2005). Envelope protein requirements for the assembly of infectious virions of porcine reproductive and respiratory syndrome virus. J. Virol..

[32] Dörner T., Radbruch A. (2007). Antibodies and B cell memory in viral immunity. Immunity.

[33] Young C., Brink R. (2021). The unique biology of germinal center B cells. Immunity.

[34] Nelson E.A., Christopher-Hennings J., Benfield D.A. (1994). Serum immune responses to the proteins of porcine reproductive and respiratory syndrome (PRRS) virus. J. Vet. Diagn. Investig..

[35] Yoon I.J., Joo H.S., Goyal S.M., Molitor T.W. (1994). A modified serum neutralization test for the detection of antibody to porcine reproductive and respiratory syndrome virus in swine sera. J. Vet. Diagn. Investig..

[36] Díaz I., Darwich L., Pappaterra G., Pujols J., Mateu E. (2005). Immune responses of pigs after experimental infection with a European strain of Porcine reproductive and respiratory syndrome virus. J. Gen. Virol..

[37] Rossow K.D., Bautista E.M., Goyal S.M., Molitor T.W., Murtaugh M.P., Morrison R.B., Benfield D.A., Collins J.E. (1994). Experimental porcine reproductive and respiratory syndrome virus infection in one-, four-, and 10-week-old pigs. J. Vet. Diagn. Investig..

[38] van der Linden I.F.A., Voermans J.J.M., van der Linde-Bril E.M., Bianchi A.T.J., Steverink P.J.G.M. (2003). Virological kinetics and immunological responses to a porcine reproductive and respiratory syndrome virus infection of pigs at different ages. Vaccine.

[39] Loemba H.D., Mounir S., Mardassi H., Archambault D., Dea S. (1996). Kinetics of humoral immune response to the major structural proteins of the porcine reproductive and respiratory syndrome virus. Arch. Virol..

[40] Yoon K.J., Zimmerman J.J., Swenson S.L., McGinley M.J., Eernisse K.A., Brevik A., Rhinehart L.L., Frey M.L., Hill H.T., Platt K.B. (1995). Characterization of the humoral immune response to porcine reproductive and respiratory syndrome (PRRS) virus infection. J. Vet. Diagn. Investig..

[41] Loving C.L., Osorio F.A., Murtaugh M.P., Zuckermann F.A. (2015). Innate and adaptive immunity against Porcine Reproductive and Respiratory Syndrome Virus. Vet. Immunol. Immunopathol..

[42] Bocard L.V., Kick A.R., Hug C., Lischer H.E.L., Käser T., Summerfield A. (2021). Systems Immunology Analyses Following Porcine Respiratory and Reproductive Syndrome Virus Infection and Vaccination. Front. Immunol..

[43] Butler J.E., Wertz N., Weber P., Lager K.M. (2008). Porcine reproductive and respiratory syndrome virus subverts repertoire development by proliferation of germline-encoded B cells of all isotypes bearing hydrophobic heavy chain CDR3. J. Immunol..

[44] Osorio F.A., Galeota J.A., Nelson E., Brodersen B., Doster A., Wills R., Zuckermann F., Laegreid W.W. (2002). Passive transfer of virus-specific antibodies confers protection against reproductive failure induced by a virulent strain of porcine reproductive and respiratory syndrome virus and establishes sterilizing immunity. Virology.

[45] Lopez O.J., Oliveira M.F., Garcia E.A., Kwon B.J., Doster A., Osorio F.A. (2007). Protection against porcine reproductive and respiratory syndrome virus (PRRSV) infection through passive transfer of PRRSV-neutralizing antibodies is dose dependent. Clin. Vaccine Immunol..

[46] Robinson S.R., Rahe M.C., Gray D.K., Martins K.V., Murtaugh M.P. (2018). Porcine reproductive and respiratory syndrome virus neutralizing antibodies provide in vivo cross-protection to PRRSV1 and PRRSV2 viral challenge. Virus Res..

[47] Ostrowski M., Galeota J.A., Jar A.M., Platt K.B., Osorio F.A., Lopez O.J. (2002). Identification of neutralizing and nonneutralizing epitopes in the porcine reproductive and respiratory syndrome virus GP5 ectodomain. J. Virol..

[48] Pirzadeh B., Dea S. (1998). Immune response in pigs vaccinated with plasmid DNA encoding ORF5 of porcine reproductive and respiratory syndrome virus. J. Gen. Virol..

[49] Jiang Y., Xiao S., Fang L., Yu X., Song Y., Niu C., Chen H. (2006). DNA vaccines co-expressing GP5 and M proteins of porcine reproductive and respiratory syndrome virus (PRRSV) display enhanced immunogenicity. Vaccine.

[50] Fan B., Liu X., Bai J., Zhang T., Zhang Q., Jiang P. (2016). Influence of the amino acid residues at 70 in M protein of porcine reproductive and respiratory syndrome virus on viral neutralization susceptibility to the serum antibody. Virol. J..

[51] Fan B., Liu X., Bai J., Zhang T., Zhang Q., Jiang P. (2015). The amino acid residues at 102 and 104 in GP5 of porcine reproductive and respiratory syndrome virus regulate viral neutralization susceptibility to the porcine serum neutralizing antibody. Virus Res..

[52] Das P.B., Dinh P.X., Ansari I.H., de Lima M., Osorio F.A., Pattnaik A.K. (2010). The minor envelope glycoproteins GP2a and GP4 of porcine reproductive and respiratory syndrome virus interact with the receptor CD163. J. Virol..

[53] Kimpston-Burkgren K., Correas I., Osorio F.A., Steffen D., Pattnaik A.K., Fang Y., Vu H.L.X. (2017). Relative contribution of porcine reproductive and respiratory syndrome virus open reading frames 2-4 to the induction of protective immunity. Vaccine.

[54] Vanhee M., Van Breedam W., Costers S., Geldhof M., Noppe Y., Nauwynck H. (2011). Characterization of antigenic regions in the porcine reproductive and respiratory syndrome virus by the use of peptide-specific serum antibodies. Vaccine.

[55] Meulenberg J.J., van Nieuwstadt A.P., van Essen-Zandbergen A., Langeveld J.P. (1997). Posttranslational processing and identification of a neutralization domain of the GP4 protein encoded by ORF4 of Lelystad virus. J. Virol..

[56] Chen X., Zhao J., Ji P., Li X., Niu H., Jiao D., Zhang L., Zhu Q., Liu X., Hiscox J.A. (2025). A Porcine Monoclonal Antibody Targeting a Conserved GP4 Epitope Protects against In Vivo Infection via the Induction of Broad-Spectrum PRRSV Neutralization. Adv. Sci..

[57] Veselkova B., Saikia C., Affeldt S., Barth S., Rajak M., Besa F., Bezbakh D., Hansen G., Rümenapf T., Ballmaier T. (2026). Structural basis of porcine reproductive and respiratory syndrome virus 2 neutralization by a GP4-targeting monoclonal antibody. J. Gen. Virol..

[58] Vu H.L., Kwon B., Yoon K.J., Laegreid W.W., Pattnaik A.K., Osorio F.A. (2011). Immune evasion of porcine reproductive and respiratory syndrome virus through glycan shielding involves both glycoprotein 5 as well as glycoprotein 3. J. Virol..

[59] Wang T.Y., Sun M.X., Zhang H.L., Wang G., Zhan G., Tian Z.J., Cai X.H., Su C., Tang Y.D. (2021). Evasion of Antiviral Innate Immunity by Porcine Reproductive and Respiratory Syndrome Virus. Front. Microbiol..

[60] Chen X.X., Qiao S., Li R., Wang J., Li X., Zhang G. (2023). Evasion strategies of porcine reproductive and respiratory syndrome virus. Front. Microbiol..

[61] Duan X., Nauwynck H.J., Pensaert M.B. (1997). Virus quantification and identification of cellular targets in the lungs and lymphoid tissues of pigs at different time intervals after inoculation with porcine reproductive and respiratory syndrome virus (PRRSV). Vet. Microbiol..

[62] Lunney J.K., Fang Y., Ladinig A., Chen N., Li Y., Rowland B., Renukaradhya G.J. (2016). Porcine Reproductive and Respiratory Syndrome Virus (PRRSV): Pathogenesis and Interaction with the Immune System. Annu. Rev. Anim. Biosci..

[63] Butler J.E., Lager K.M., Golde W., Faaberg K.S., Sinkora M., Loving C., Zhang Y.I. (2014). Porcine reproductive and respiratory syndrome (PRRS): An immune dysregulatory pandemic. Immunol. Res..

[64] Wang R., Nan Y., Yu Y., Zhang Y.J. (2013). Porcine reproductive and respiratory syndrome virus Nsp1β inhibits interferon-activated JAK/STAT signal transduction by inducing karyopherin-α1 degradation. J. Virol..

[65] Sun Y., Ke H., Han M., Chen N., Fang W., Yoo D. (2016). Nonstructural Protein 11 of Porcine Reproductive and Respiratory Syndrome Virus Suppresses Both MAVS and RIG-I Expression as One of the Mechanisms to Antagonize Type I Interferon Production. PLoS ONE.

[66] Burgara-Estrella A., Díaz I., Rodríguez-Gómez I.M., Essler S.E., Hernández J., Mateu E. (2013). Predicted peptides from non-structural proteins of porcine reproductive and respiratory syndrome virus are able to induce IFN-γ and IL-10. Viruses.

[67] Wongyanin P., Buranapraditkul S., Yoo D., Thanawongnuwech R., Roth J.A., Suradhat S. (2012). Role of porcine reproductive and respiratory syndrome virus nucleocapsid protein in induction of interleukin-10 and regulatory T-lymphocytes (Treg). J. Gen. Virol..

[68] Li Y., Mateu E. (2026). TNF-α and IL-10 differentially modulate apoptosis during PRRSV-1 infection of bone marrow-derived dendritic cells. BMC Vet. Res..

[69] Singleton H., Graham S.P., Frossard J.P., Bodman-Smith K.B., Steinbach F. (2018). Infection of monocytes with European porcine reproductive and respiratory syndrome virus (PRRSV-1) strain Lena is significantly enhanced by dexamethasone and IL-10. Virology.

[70] Flores-Mendoza L., Silva-Campa E., Reséndiz M., Osorio F.A., Hernández J. (2008). Porcine reproductive and respiratory syndrome virus infects mature porcine dendritic cells and up-regulates interleukin-10 production. Clin. Vaccine Immunol..

[71] Wang G., Yu Y., Cai X., Zhou E.M., Zimmerman J.J. (2020). Effects of PRRSV Infection on the Porcine Thymus. Trends Microbiol..

[72] Cerrato S., Brazis P., Valle M.F.D., Miolo A., Puigdemont A. (2009). Veterinary Immunology and Immunopathology. Vet. Immunol. Immunopathol..

[73] Molina R.M., Cha S.H., Chittick W., Lawson S., Murtaugh M.P., Nelson E.A., Christopher-Hennings J., Yoon K.J., Evans R., Rowland R.R. (2008). Immune response against porcine reproductive and respiratory syndrome virus during acute and chronic infection. Vet. Immunol. Immunopathol..

[74] Vézina S.A., Loemba H., Fournier M., Dea S., Archambault D. (1996). Antibody production and blastogenic response in pigs experimentally infected with porcine reproductive and respiratory syndrome virus. Can. J. Vet. Res..

[75] Abdel-Hafeez E.H., Watanabe K., Kamei K., Kikuchi M., Chen H., Daniel B., Yu C., Hirayama K. (2014). Pilot Study on Interferon-γ-producing T Cell Subsets after the Protective Vaccination with Radiation-attenuated Cercaria of Schistosoma japonicum in the Miniature Pig Model. Trop. Med. Health.

[76] Ciofani M., Zúñiga-Pflücker J.C. (2010). Determining γδ versus α T cell development. Nat. Rev. Immunol..

[77] Gerner W., Mair K.H., Schmidt S. (2022). Local and Systemic T Cell Immunity in Fighting Pig Viral and Bacterial Infections. Annu. Rev. Anim. Biosci..

[78] Pescovitz M.D., Sakopoulos A.G., Gaddy J.A., Husmann R.J., Zuckermann F.A. (1994). Porcine peripheral blood CD4+/CD8+ dual expressing T cells. Vet. Immunol. Immunopathol..

[79] Zuckermann F.A., Husmann R.J. (1996). Functional and phenotypic analysis of porcine peripheral blood CD4/CD8 double-positive T cells. Immunology.

[80] Hu Y., Hu Q., Li Y., Lu L., Xiang Z., Yin Z., Kabelitz D., Wu Y. (2023). γδ T cells: Origin and fate, subsets, diseases and immunotherapy. Signal Transduct. Target. Ther..

[81] Bettin L., Darbellay J., van Kessel J., Dhar N., Gerdts V. (2024). Porcine γδ T cells express cytotoxic cell-associated markers and display killing activity but are not selectively cytotoxic against PRRSV- or swIAV-infected macrophages. Front. Immunol..

[82] Beringer D.X., Straetemans T., Minguet S., Riillo C., Lynch L., Sebestyen Z., Kuball J. (2025). Disrupting the balance between activating and inhibitory receptors of γδT cells for effective cancer immunotherapy. Nat. Rev. Cancer.

[83] Nielsen J., Bøtner A. (1997). Hematological and immunological parameters of 4 1/2-month old pigs infected with PRRS virus. Vet. Microbiol..

[84] Samsom J.N., de Bruin T.G., Voermans J.J., Meulenberg J.J., Pol J.M., Bianchi A.T. (2000). Changes of leukocyte phenotype and function in the broncho-alveolar lavage fluid of pigs infected with porcine reproductive and respiratory syndrome virus: A role for CD8(+) cells. J. Gen. Virol..

[85] Kick A.R., Amaral A.F., Cortes L.M., Fogle J.E., Crisci E., Almond G.W., Käser T. (2019). The T cell Response to Type 2 Porcine Reproductive and Respiratory Syndrome Virus (PRRSV). Viruses.

[86] Xiao Z., Batista L., Dee S., Halbur P., Murtaugh M.P. (2004). The level of virus-specific T cell and macrophage recruitment in porcine reproductive and respiratory syndrome virus infection in pigs is independent of virus load. J. Virol..

[87] Li Z., He Y., Xu X., Leng X., Li S., Wen Y., Wang F., Xia M., Cheng S., Wu H. (2016). Pathological and immunological characteristics of piglets infected experimentally with a HP-PRRSV TJ strain. BMC Vet. Res..

[88] Zhang W., Wang X., Zhang H., Pan Y., Ma W., Xu Y., Tian Z., Xia C., Fu L., Wang Y. (2024). Comparison of pathogenicity and host responses of emerging porcine reproductive and respiratory syndrome virus variants in piglets. J. Virol..

[89] Ferrari L., Canelli E., De Angelis E., Catella A., Ferrarini G., Ogno G., Bonati L., Nardini R., Borghetti P., Martelli P. (2018). A highly pathogenic porcine reproductive and respiratory syndrome virus type 1 (PRRSV-1) strongly modulates cellular innate and adaptive immune subsets upon experimental infection. Vet. Microbiol..

[90] Nazki S., Khatun A., Jeong C.G., Mattoo S.U.S., Gu S., Lee S.I., Kim S.C., Park J.H., Yang M.S., Kim B. (2020). Evaluation of local and systemic immune responses in pigs experimentally challenged with porcine reproductive and respiratory syndrome virus. Vet. Res..

[91] Akdis C.A. (2009). T cells in health and disease. J. Allergy Clin. Immunol..

[92] Schulz E.G., Mariani L., Radbruch A., Höfer T. (2009). Sequential polarization and imprinting of type 1 T helper lymphocytes by interferon-gamma and interleukin-12. Immunity.

[93] Ebner F., Rausch S., Scharek-Tedin L., Pieper R., Burwinkel M., Zentek J., Hartmann S. (2014). A novel lineage transcription factor based analysis reveals differences in T helper cell subpopulation development in infected and intrauterine growth restricted (IUGR) piglets. Dev. Comp. Immunol..

[94] Fuertes L., Doménech N., Alvarez B., Ezquerra A., Domínguez J., Castro J.M., Alonso F. (1999). Analysis of cellular immune response in pigs recovered from porcine respiratory and reproductive syndrome infection. Virus Res..

[95] Mills K.H.G. (2023). IL-17 and IL-17-producing cells in protection versus pathology. Nat. Rev. Immunol..

[96] Zhang L., Zhou L., Ge X., Guo X., Han J., Yang H. (2016). The Chinese highly pathogenic porcine reproductive and respiratory syndrome virus infection suppresses Th17 cells response in vivo. Vet. Microbiol..

[97] Song W., Craft J. (2024). T Follicular Helper Cell Heterogeneity. Annu. Rev. Immunol..

[98] Li S., Qiu M., Li S., Li C., Lin H., Qiu Y., Qi W., Feng B., Cui M., Yang S. (2024). A chimeric porcine reproductive and respiratory syndrome virus 1 strain containing synthetic ORF2-6 genes can trigger T follicular helper cell and heterologous neutralizing antibody responses and confer enhanced cross-protection. Vet. Res..

[99] Qiu M., Li S., Cui M., Yang S., Zhu X., Meng Y., Lin Y., Lin H., Sun Z., Wang Y. (2025). Efficacy of a non-recombinant NADC34-like PRRSV-2 vaccine candidate against a recombinant NADC34-like PRRSV-2 isolate. Vet. Microbiol..

[100] Guo S., Wang X., Zhu J., Liu C., Zhou H., Chen Y.-Q., Bai Y., Liu H.-R., Feng W.-H. (2026). Single-cell transcriptomic profiling of bronchial lymph nodes reveals mechanisms of PRRSV escape from host adaptive immunity. Front. Immunol..

[101] Goldmann O., Nwofor O.V., Chen Q., Medina E. (2024). Mechanisms underlying immunosuppression by regulatory cells. Front. Immunol..

[102] Vignali D.A., Collison L.W., Workman C.J. (2008). How regulatory T cells work. Nat. Rev. Immunol..

[103] Bolzer K., Käser T., Saalmüller A., Hammer S.E. (2009). Molecular characterisation of porcine Forkhead-box p3 (Foxp3). Vet. Immunol. Immunopathol..

[104] Käser T., Gerner W., Saalmüller A. (2011). Porcine regulatory T cells: Mechanisms and T cell targets of suppression. Dev. Comp. Immunol..

[105] Silva-Campa E., Mata-Haro V., Mateu E., Hernández J. (2012). Porcine reproductive and respiratory syndrome virus induces CD4+CD8+CD25+Foxp3+ regulatory T cells (Tregs). Virology.

[106] Silva-Campa E., Flores-Mendoza L., Reséndiz M., Pinelli-Saavedra A., Mata-Haro V., Mwangi W., Hernández J. (2009). Induction of T helper 3 regulatory cells by dendritic cells infected with porcine reproductive and respiratory syndrome virus. Virology.

[107] Silva-Campa E., Cordoba L., Fraile L., Flores-Mendoza L., Montoya M., Hernández J. (2010). European genotype of porcine reproductive and respiratory syndrome (PRRSV) infects monocyte-derived dendritic cells but does not induce Treg cells. Virology.

[108] Nedumpun T., Sirisereewan C., Thanmuan C., Techapongtada P., Puntarotairung R., Naraprasertkul S., Thanawongnuwech R., Suradhat S. (2018). Induction of porcine reproductive and respiratory syndrome virus (PRRSV)-specific regulatory T lymphocytes (Treg) in the lungs and tracheobronchial lymph nodes of PRRSV-infected pigs. Vet. Microbiol..

[109] Ruedas-Torres I., Gómez-Laguna J., Sánchez-Carvajal J.M., Larenas-Muñoz F., Barranco I., Pallarés F.J., Carrasco L., Rodríguez-Gómez I.M. (2021). Activation of T-bet, FOXP3, and EOMES in Target Organs From Piglets Infected With the Virulent PRRSV-1 Lena Strain. Front. Immunol..

[110] Costers S., Lefebvre D.J., Goddeeris B., Delputte P.L., Nauwynck H.J. (2009). Functional impairment of PRRSV-specific peripheral CD3+CD8high cells. Vet. Res..

[111] Appay V., Yamamoto T., Sáez-Cirión A. (2026). Renaissance of antiviral CD8(+) T cell immunity in vaccination and disease. Nat. Rev. Immunol..

[112] Chung C.J., Cha S.H., Grimm A.L., Ajithdoss D., Rzepka J., Chung G., Yu J., Davis W.C., Ho C.S. (2018). Pigs that recover from porcine reproduction and respiratory syndrome virus infection develop cytotoxic CD4+CD8+ and CD4+CD8- T cells that kill virus infected cells. PLoS ONE.

[113] Li Y., Diaz I., Martin-Valls G., Beyersdorf N., Mateu E. (2023). Systemic CD4 cytotoxic T cells improve protection against PRRSV-1 transplacental infection. Front. Immunol..

[114] Olin M.R., Batista L., Xiao Z., Dee S.A., Murtaugh M.P., Pijoan C.C., Molitor T.W. (2005). Gammadelta lymphocyte response to porcine reproductive and respiratory syndrome virus. Viral Immunol..

[115] Hu W., Tang D., Zeng Z., Wang B., Zhou M., Mao Y., Zhou P., He S. (2025). Research progress on the molecular mechanism of immune escape of porcine reproductive and respiratory syndrome virus. Virology.

[116] Harding C.V. (1991). Pathways of antigen processing. Curr. Opin. Immunol..

[117] Hammer S.E., Ho C.S., Ando A., Rogel-Gaillard C., Charles M., Tector M., Tector A.J., Lunney J.K. (2020). Importance of the Major Histocompatibility Complex (Swine Leukocyte Antigen) in Swine Health and Biomedical Research. Annu. Rev. Anim. Biosci..

[118] Lunney J.K., Ho C.S., Wysocki M., Smith D.M. (2009). Molecular genetics of the swine major histocompatibility complex, the SLA complex. Dev. Comp. Immunol..

[119] Cai H., Zhang H., Cheng H., Liu M., Wen S., Ren J. (2023). Progress in PRRSV Infection and Adaptive Immune Response Mechanisms. Viruses.

[120] Rodríguez-Gómez I.M., Gómez-Laguna J., Carrasco L. (2013). Impact of PRRSV on activation and viability of antigen presenting cells. World J. Virol..

[121] Mötz M., Stas M.R., Hammer S.E., Duckova T., Fontaine F., Kiesler A., Seitz K., Ladinig A., Müller A.C., Riedel C. (2022). Identification of MHC-I-Presented Porcine Respiratory and Reproductive Syndrome Virus (PRRSV) Peptides Reveals Immunogenic Epitopes within Several Non-Structural Proteins Recognized by CD8(+) T Cells. Viruses.

[122] Liu Y., Gao P., Zhou L., Ge X., Zhang Y., Guo X., Han J., Yang H. (2022). Mapping the Key Residues within the Porcine Reproductive and Respiratory Syndrome Virus nsp1α Replicase Protein Required for Degradation of Swine Leukocyte Antigen Class I Molecules. Viruses.

[123] Cao Q.M., Subramaniam S., Ni Y.Y., Cao D., Meng X.J. (2016). The non-structural protein Nsp2TF of porcine reproductive and respiratory syndrome virus down-regulates the expression of Swine Leukocyte Antigen class I. Virology.

[124] Du J., Ge X., Liu Y., Jiang P., Wang Z., Zhang R., Zhou L., Guo X., Han J., Yang H. (2016). Targeting Swine Leukocyte Antigen Class I Molecules for Proteasomal Degradation by the nsp1α Replicase Protein of the Chinese Highly Pathogenic Porcine Reproductive and Respiratory Syndrome Virus Strain JXwn06. J. Virol..

[125] Kang L., Wahaab A., Qi P., Qiu Y., Wei J., Li B., Shao D., Li Z., Liu K., Ma Z. (2024). Porcine reproductive and respiratory syndrome virus nsp4-mediated β2M downregulation contributes to SLA-I decrease and virus infection in vivo and in vitro. Virology.

[126] Chen X., Bai J., Liu X., Song Z., Zhang Q., Wang X., Jiang P. (2018). Nsp1α of Porcine Reproductive and Respiratory Syndrome Virus Strain BB0907 Impairs the Function of Monocyte-Derived Dendritic Cells via the Release of Soluble CD83. J. Virol..

[127] Rodríguez-Gómez I.M., Käser T., Gómez-Laguna J., Lamp B., Sinn L., Rümenapf T., Carrasco L., Saalmüller A., Gerner W. (2015). PRRSV-infected monocyte-derived dendritic cells express high levels of SLA-DR and CD80/86 but do not stimulate PRRSV-naïve regulatory T cells to proliferate. Vet. Res..

[128] Wu C., Shi B., Yang D., Zhang K., Li J., Wang J., Liu H., Zhao Q., Zhou E.M., Nan Y. (2020). Porcine Reproductive and Respiratory Syndrome Virus Promotes SLA-DR-Mediated Antigen Presentation of Nonstructural Proteins To Evoke a Nonneutralizing Antibody Response In Vivo. J. Virol..

[129] Ruedas-Torres I., Rodríguez-Gómez I.M., Sánchez-Carvajal J.M., Guil-Luna S., Larenas-Muñoz F., Pallarés F.J., Carrasco L., Gómez-Laguna J. (2021). Up-Regulation of Immune Checkpoints in the Thymus of PRRSV-1-Infected Piglets in a Virulence-Dependent Fashion. Front. Immunol..

[130] Cox M.A., Nechanitzky R., Mak T.W. (2017). Check point inhibitors as therapies for infectious diseases. Curr. Opin. Immunol..

[131] Sun Q., Dong C. (2026). Regulators of CD8(+) T cell exhaustion. Nat. Rev. Immunol..

[132] Ruedas-Torres I., Sánchez-Carvajal J.M., Carrasco L., Pallarés F.J., Larenas-Muñoz F., Rodríguez-Gómez I.M., Gómez-Laguna J. (2022). PRRSV-1 induced lung lesion is associated with an imbalance between costimulatory and coinhibitory immune checkpoints. Front. Microbiol..

[133] Chaudhari J., Liew C.S., Workman A.M., Riethoven J.M., Steffen D., Sillman S., Vu H.L.X. (2020). Host Transcriptional Response to Persistent Infection with a Live-Attenuated Porcine Reproductive and Respiratory Syndrome Virus Strain. Viruses.

[134] Sánchez-Carvajal J.M., Rodríguez-Gómez I.M., Ruedas-Torres I., Zaldívar-López S., Larenas-Muñoz F., Bautista-Moreno R., Garrido J.J., Pallarés F.J., Carrasco L., Gómez-Laguna J. (2022). Time Series Transcriptomic Analysis of Bronchoalveolar Lavage Cells from Piglets Infected with Virulent or Low-Virulent Porcine Reproductive and Respiratory Syndrome Virus 1. J. Virol..

[135] Lim B., Kim S.C., Kim H.J., Kim J.H., Seo Y.J., Lim C., Park Y., Sheet S., Kim D., Lim D.H. (2025). Single-cell transcriptomics of bronchoalveolar lavage during PRRSV infection with different virulence. Nat. Commun..

[136] Kim S.C., Kim H.J., Kang S.C., Rasool A., Ryu J.H., Lee J.M., Kim W.I. (2025). The role of immune checkpoint molecules in PRRSV-2-induced immune modulation: Insights from comparative in vivo evaluation including NADC34-like PRRSV. J. Virol..

[137] Wang H., Zheng X., Zheng D., Wang X., Zhao Z., Zhao M., Guo Q., Mu Y. (2024). Monoclonal Antibody against Porcine LAG3 Inhibits Porcine Reproductive and Respiratory Syndrome Virus Infection. Vet. Sci..

[138] Martelli P., Gozio S., Ferrari L., Rosina S., De Angelis E., Quintavalla C., Bottarelli E., Borghetti P. (2009). Efficacy of a modified live porcine reproductive and respiratory syndrome virus (PRRSV) vaccine in pigs naturally exposed to a heterologous European (Italian cluster) field strain: Clinical protection and cell-mediated immunity. Vaccine.

[139] Rose N., Renson P., Andraud M., Paboeuf F., Le Potier M.F., Bourry O. (2015). Porcine reproductive and respiratory syndrome virus (PRRSv) modified-live vaccine reduces virus transmission in experimental conditions. Vaccine.

[140] Mou C., Zhao X., Zhuo C., He Q., Xu M., Shi K., Han T., Xu S., Chen Z. (2025). The mRNA vaccine expressing fused structural protein of PRRSV protects piglets against PRRSV challenge. Vet. Microbiol..

[141] Chang X., Ma J., Zhou Y., Xiao S., Xiao X., Fang L. (2024). Development of a Ferritin Protein Nanoparticle Vaccine with PRRSV GP5 Protein. Viruses.

[142] Xie C., Ha Z., Sun W., Nan F., Zhang P., Han J., Zhao G., Zhang H., Zhuang X., Lu H. (2019). Construction and immunological evaluation of recombinant adenovirus vaccines co-expressing GP3 and GP5 of EU-type porcine reproductive and respiratory syndrome virus in pigs. J. Vet. Med. Sci..

[143] Oh T., Kim H., Park K.H., Jeong J., Kang I., Yang S., Chae C. (2019). Effectiveness of a commercial porcine reproductive and respiratory syndrome virus (PRRSV) subunit vaccine against heterologous PRRSV-1 and PRRSV-2 challenge in late-term pregnant gilts. Can. J. Vet. Res..

[144] Lunney J.K., Benfield D.A., Rowland R.R. (2010). Porcine reproductive and respiratory syndrome virus: An update on an emerging and re-emerging viral disease of swine. Virus Res..

[145] Murtaugh M.P., Stadejek T., Abrahante J.E., Lam T.T., Leung F.C. (2010). The ever-expanding diversity of porcine reproductive and respiratory syndrome virus. Virus Res..

[146] Kappes M.A., Faaberg K.S. (2015). PRRSV structure, replication and recombination: Origin of phenotype and genotype diversity. Virology.

[147] Murtaugh M.P., Genzow M. (2011). Immunological solutions for treatment and prevention of porcine reproductive and respiratory syndrome (PRRS). Vaccine.

[148] Raev S.A., Cai L., Muro N., Madera R., Wang L., Shi J. (2026). Cross-Protection in PRRSV: Mechanisms, Limitations, and Implications for Vaccine Design. Pathogens.

[149] Kick A.R., Grete A.F., Crisci E., Almond G.W., Käser T. (2023). Testable Candidate Immune Correlates of Protection for Porcine Reproductive and Respiratory Syndrome Virus Vaccination. Vaccines.

[150] Nan Y., Wu C., Gu G., Sun W., Zhang Y.J., Zhou E.M. (2017). Improved Vaccine against PRRSV: Current Progress and Future Perspective. Front. Microbiol..

[151] Renukaradhya G.J., Meng X.J., Calvert J.G., Roof M., Lager K.M. (2015). Live porcine reproductive and respiratory syndrome virus vaccines: Current status and future direction. Vaccine.

[152] Chae C. (2021). Commercial PRRS Modified-Live Virus Vaccines. Vaccines.

[153] Wang J., Zhang M., Cui X., Gao X., Sun W., Ge X., Zhang Y., Guo X., Han J., Zhou L. (2022). Attenuated Porcine Reproductive and Respiratory Syndrome Virus Regains Its Fatal Virulence by Serial Passaging in Pigs or Porcine Alveolar Macrophages To Increase Its Adaptation to Target Cells. Microbiol. Spectr..

[154] Proctor J., Wolf I., Brodsky D., Cortes L.M., Frias-De-Diego A., Almond G.W., Crisci E., Negrão Watanabe T.T., Hammer J.M., Käser T. (2022). Heterologous vaccine immunogenicity, efficacy, and immune correlates of protection of a modified-live virus porcine reproductive and respiratory syndrome virus vaccine. Front. Microbiol..

[155] Charerntantanakul W., Platt R., Johnson W., Roof M., Vaughn E., Roth J.A. (2006). Immune responses and protection by vaccine and various vaccine adjuvant candidates to virulent porcine reproductive and respiratory syndrome virus. Vet. Immunol. Immunopathol..

[156] Stadler J., Naderer L., Beffort L., Ritzmann M., Emrich D., Hermanns W., Fiebig K., Saalmüller A., Gerner W., Glatthaar-Saalmüller B. (2021). Safety and immune responses after intradermal application of Porcilis PRRS in either the neck or the perianal region. PLoS ONE.

[157] Madapong A., Saeng-Chuto K., Boonsoongnern A., Tantituvanont A., Nilubol D. (2020). Cell-mediated immune and protective efficacy of porcine reproductive and respiratory syndrome virus modified-live vaccines against co-challenge with PRRSV-1 and PRRSV-2. Sci. Rep..

[158] Vu H.L., Ma F., Laegreid W.W., Pattnaik A.K., Steffen D., Doster A.R., Osorio F.A. (2015). A Synthetic Porcine Reproductive and Respiratory Syndrome Virus Strain Confers Unprecedented Levels of Heterologous Protection. J. Virol..

[159] Li X., Galliher-Beckley A., Pappan L., Trible B., Kerrigan M., Beck A., Hesse R., Blecha F., Nietfeld J.C., Rowland R.R. (2014). Comparison of host immune responses to homologous and heterologous type II porcine reproductive and respiratory syndrome virus (PRRSV) challenge in vaccinated and unvaccinated pigs. Biomed. Res. Int..

[160] Park C., Seo H.W., Han K., Kang I., Chae C. (2014). Evaluation of the efficacy of a new modified live porcine reproductive and respiratory syndrome virus (PRRSV) vaccine (Fostera PRRS) against heterologous PRRSV challenge. Vet. Microbiol..

[161] Hou F.H., Lee W.C., Liao J.W., Chien M.S., Kuo C.J., Chung H.P., Chia M.Y. (2020). Evaluation of a type 2 modified live porcine reproductive and respiratory syndrome vaccine against heterologous challenge of a lineage 3 highly virulent isolate in pigs. PeerJ.

[162] Savard C., Alvarez F., Provost C., Chorfi Y., D’Allaire S., Benoit-Biancamano M.O., Gagnon C.A. (2016). Efficacy of Fostera PRRS modified live virus vaccine against a Canadian heterologous virulent field strain of porcine reproductive and respiratory syndrome virus. Can. J. Vet. Res..

[163] Canelli E., Catella A., Borghetti P., Ferrari L., Ogno G., De Angelis E., Bonilauri P., Guazzetti S., Nardini R., Martelli P. (2018). Efficacy of a modified-live virus vaccine in pigs experimentally infected with a highly pathogenic porcine reproductive and respiratory syndrome virus type 1 (HP-PRRSV-1). Vet. Microbiol..

[164] Jeong J., Kim S., Park K.H., Kang I., Park S.J., Park C., Chae C. (2017). Evaluation of the effect of a porcine reproductive and respiratory syndrome (PRRS) modified-live virus vaccine on sow reproductive performance in endemic PRRS farms. Vet. Microbiol..

[165] Pileri E., Gibert E., Soldevila F., García-Saenz A., Pujols J., Diaz I., Darwich L., Casal J., Martín M., Mateu E. (2015). Vaccination with a genotype 1 modified live vaccine against porcine reproductive and respiratory syndrome virus significantly reduces viremia, viral shedding and transmission of the virus in a quasi-natural experimental model. Vet. Microbiol..

[166] Renson P., Fablet C., Andraud M., Normand V., Lebret A., Paboeuf F., Rose N., Bourry O. (2019). Maternally-derived neutralizing antibodies reduce vaccine efficacy against porcine reproductive and respiratory syndrome virus infection. Vaccine.

[167] Lakshmanappa Y.S., Shang P., Renu S., Dhakal S., Hogshead B., Xiao Y., Wang T., Fang Y., Renukaradhya G.J. (2025). Concurrent but consecutive vaccination of modified live PRRSV-1 and PRRSV-2 provides better protection in nursery pigs. Vet. Microbiol..

[168] Chase-Topping M., Xie J., Pooley C., Trus I., Bonckaert C., Rediger K., Bailey R.I., Brown H., Bitsouni V., Barrio M.B. (2020). New insights about vaccine effectiveness: Impact of attenuated PRRS-strain vaccination on heterologous strain transmission. Vaccine.

[169] Kim T., Park C., Choi K., Jeong J., Kang I., Park S.J., Chae C. (2015). Comparison of Two Commercial Type 1 Porcine Reproductive and Respiratory Syndrome Virus (PRRSV) Modified Live Vaccines against Heterologous Type 1 and Type 2 PRRSV Challenge in Growing Pigs. Clin. Vaccine Immunol..

[170] Jeong J., Park C., Oh T., Park K.H., Yang S., Kang I., Park S.J., Chae C. (2018). Cross-protection of a modified-live porcine reproductive and respiratory syndrome virus (PRRSV)-2 vaccine against a heterologous PRRSV-1 challenge in late-term pregnancy gilts. Vet. Microbiol..

[171] Paploski I.A.D., Pamornchainavakul N., Makau D.N., Rovira A., Corzo C.A., Schroeder D.C., Cheeran M.C., Doeschl-Wilson A., Kao R.R., Lycett S. (2021). Phylogenetic Structure and Sequential Dominance of Sub-Lineages of PRRSV Type-2 Lineage 1 in the United States. Vaccines.

[172] Prieto C., Alvarez E., Martínez-Lobo F.J., Simarro I., Castro J.M. (2008). Similarity of European porcine reproductive and respiratory syndrome virus strains to vaccine strain is not necessarily predictive of the degree of protective immunity conferred. Vet. J..

[173] Díaz I., Gimeno M., Darwich L., Navarro N., Kuzemtseva L., López S., Galindo I., Segalés J., Martín M., Pujols J. (2012). Characterization of homologous and heterologous adaptive immune responses in porcine reproductive and respiratory syndrome virus infection. Vet. Res..

[174] Martínez-Lobo F.J., Díez-Fuertes F., Simarro I., Castro J.M., Prieto C. (2021). The Ability of Porcine Reproductive and Respiratory Syndrome Virus Isolates to Induce Broadly Reactive Neutralizing Antibodies Correlates With In Vivo Protection. Front. Immunol..

[175] Zhou L., Ge X., Yang H. (2021). Porcine Reproductive and Respiratory Syndrome Modified Live Virus Vaccine: A "Leaky" Vaccine with Debatable Efficacy and Safety. Vaccines.

[176] Martínez-Lobo F.J., de Lome L.C., Díez-Fuertes F., Segalés J., García-Artiga C., Simarro I., Castro J.M., Prieto C. (2013). Safety of Porcine Reproductive and Respiratory Syndrome Modified Live Virus (MLV) vaccine strains in a young pig infection model. Vet. Res..

[177] Christopher-Hennings J., Nelson E.A., Nelson J.K., Benfield D.A. (1997). Effects of a modified-live virus vaccine against porcine reproductive and respiratory syndrome in boars. Am. J. Vet. Res..

[178] Madapong A., Saeng-Chuto K., Tantituvanont A., Nilubol D. (2021). Safety of PRRSV-2 MLV vaccines administrated via the intramuscular or intradermal route and evaluation of PRRSV transmission upon needle-free and needle delivery. Sci. Rep..

[179] Bøtner A., Strandbygaard B., Sørensen K.J., Have P., Madsen K.G., Madsen E.S., Alexandersen S. (1997). Appearance of acute PRRS-like symptoms in sow herds after vaccination with a modified live PRRS vaccine. Vet. Rec..

[180] Liu P., Bai Y., Jiang X., Zhou L., Yuan S., Yao H., Yang H., Sun Z. (2018). High reversion potential of a cell-adapted vaccine candidate against highly pathogenic porcine reproductive and respiratory syndrome. Vet. Microbiol..

[181] Eclercy J., Renson P., Lebret A., Hirchaud E., Normand V., Andraud M., Paboeuf F., Blanchard Y., Rose N., Bourry O. (2019). A Field Recombinant Strain Derived from Two Type 1 Porcine Reproductive and Respiratory Syndrome Virus (PRRSV-1) Modified Live Vaccines Shows Increased Viremia and Transmission in SPF Pigs. Viruses.

[182] Kvisgaard L.K., Kristensen C.S., Ryt-Hansen P., Pedersen K., Stadejek T., Trebbien R., Andresen L.O., Larsen L.E. (2020). A recombination between two Type 1 Porcine Reproductive and Respiratory Syndrome Virus (PRRSV-1) vaccine strains has caused severe outbreaks in Danish pigs. Transbound. Emerg. Dis..

[183] Wang A., Chen Q., Wang L., Madson D., Harmon K., Gauger P., Zhang J., Li G. (2019). Recombination between Vaccine and Field Strains of Porcine Reproductive and Respiratory Syndrome Virus. Emerg. Infect. Dis..

[184] Wu W., Lin L., Ye Z., Hou R., Zhou Q., Zhou L., Yang H. (2025). A vaccine revertant of highly pathogenic porcine reproductive and respiratory syndrome virus: Re-emergence after lurking for 12 years. Microbiol. Spectr..

[185] Lai M.M., Baric R.S., Makino S., Keck J.G., Egbert J., Leibowitz J.L., Stohlman S.A. (1985). Recombination between nonsegmented RNA genomes of murine coronaviruses. J. Virol..

[186] Wang H., Xu Y., Feng W. (2021). Porcine Reproductive and Respiratory Syndrome Virus: Immune Escape and Application of Reverse Genetics in Attenuated Live Vaccine Development. Vaccines.

[187] Chaudhari J., Vu H.L.X. (2020). Porcine Reproductive and Respiratory Syndrome Virus Reverse Genetics and the Major Applications. Viruses.

[188] Han W., Wu J.J., Deng X.Y., Cao Z., Yu X.L., Wang C.B., Zhao T.Z., Chen N.H., Hu H.H., Bin W. (2009). Molecular mutations associated with the in vitro passage of virulent porcine reproductive and respiratory syndrome virus. Virus Genes.

[189] An T.Q., Tian Z.J., Zhou Y.J., Xiao Y., Peng J.M., Chen J., Jiang Y.F., Hao X.F., Tong G.Z. (2011). Comparative genomic analysis of five pairs of virulent parental/attenuated vaccine strains of PRRSV. Vet. Microbiol..

[190] Leng X., Li Z., Xia M., Li X., Wang F., Wang W., Zhang X., Wu H. (2012). Mutations in the genome of the highly pathogenic porcine reproductive and respiratory syndrome virus potentially related to attenuation. Vet. Microbiol..

[191] Allende R., Kutish G.F., Laegreid W., Lu Z., Lewis T.L., Rock D.L., Friesen J., Galeota J.A., Doster A.R., Osorio F.A. (2000). Mutations in the genome of porcine reproductive and respiratory syndrome virus responsible for the attenuation phenotype. Arch. Virol..

[192] Li L., Chen J., Cao Z., Guo Z., Liu J., Zhou Y., Tong G., Gao F. (2025). Engineering a live-attenuated porcine reproductive and respiratory syndrome virus vaccine to prevent RNA recombination by rewiring transcriptional regulatory sequences. mBio.

[193] Hanson G., Coller J. (2018). Codon optimality, bias and usage in translation and mRNA decay. Nat. Rev. Mol. Cell Biol..

[194] Coleman J.R., Papamichail D., Skiena S., Futcher B., Wimmer E., Mueller S. (2008). Virus attenuation by genome-scale changes in codon pair bias. Science.

[195] Gao L., Wang L., Huang C., Yang L., Guo X.K., Yu Z., Liu Y., Yang P., Feng W.H. (2015). HP-PRRSV is attenuated by de-optimization of codon pair bias in its RNA-dependent RNA polymerase nsp9 gene. Virology.

[196] Ni Y.Y., Zhao Z., Opriessnig T., Subramaniam S., Zhou L., Cao D., Cao Q., Yang H., Meng X.J. (2014). Computer-aided codon-pairs deoptimization of the major envelope GP5 gene attenuates porcine reproductive and respiratory syndrome virus. Virology.

[197] Evenson D., Gerber P.F., Xiao C.T., Halbur P.G., Wang C., Tian D., Ni Y.Y., Meng X.J., Opriessnig T. (2016). A porcine reproductive and respiratory syndrome virus candidate vaccine based on the synthetic attenuated virus engineering approach is attenuated and effective in protecting against homologous virus challenge. Vaccine.

[198] Park C., Baek J.H., Cho S.H., Jeong J., Chae C., You S.H., Cha S.H. (2020). Field porcine reproductive and respiratory syndrome viruses (PRRSV) attenuated by codon pair deoptimization (CPD) in NSP1 protected pigs from heterologous challenge. Virology.

[199] Lee M.A., You S.H., Jayaramaiah U., Shin E.G., Song S.M., Ju L., Kang S.J., Cho S.H., Hyun B.H., Lee H.S. (2023). Codon Pair Deoptimization (CPD)-Attenuated PRRSV-1 Vaccination Exhibit Immunity to Virulent PRRSV Challenge in Pigs. Vaccines.

[200] Zhou L., Han J., Yang H. (2024). The evolution and diversity of porcine reproductive and respiratory syndrome virus in China. Vet. Microbiol..

[201] de Brito R.C.F., Sadigh Y., Bowman J., Clive S., Jackson B., Pedrera M., Crofts F., Bernard M., Lean F.Z.X., Núñez A. (2025). A centralised immunogen approach to develop a more broadly protective modified live porcine reproductive and respiratory syndrome virus 1 vaccine candidate. npj Vaccines.

[202] Sun H., Workman A., Osorio F.A., Steffen D., Vu H.L.X. (2018). Development of a broadly protective modified-live virus vaccine candidate against porcine reproductive and respiratory syndrome virus. Vaccine.

[203] Chen N., Li S., Tian Y., Li X., Li S., Li J., Qiu M., Sun Z., Xiao Y., Yan X. (2021). Chimeric HP-PRRSV2 containing an ORF2-6 consensus sequence induces antibodies with broadly neutralizing activity and confers cross protection against virulent NADC30-like isolate. Vet. Res..

[204] Cha S.H., Hyun B.H., Lee H.S., Kang S.J., You S.H., Jeong J., Park C.J., Lee M.S., Park C. (2023). A novel chimeric vaccine candidate for porcine reproductive and respiratory syndrome virus (PRRSV) I and II elicits neutralizing antibodies against both types. J. Gen. Virol..

[205] Li Y., Wang Y., Pei X., Chen S., Jing Y., Wu Y., Ma Z., Li Z., Zheng Z., Feng Y. (2024). A chimeric strain of porcine reproductive and respiratory syndrome virus 2 derived from HP-PRRSV and NADC30-like PRRSV confers cross-protection against both strains. Vet. Res..

[206] Pasick J. (2004). Application of DIVA vaccines and their companion diagnostic tests to foreign animal disease eradication. Anim. Health Res. Rev..

[207] van Oirschot J.T. (1999). Diva vaccines that reduce virus transmission. J. Biotechnol..

[208] de Lima M., Kwon B., Ansari I.H., Pattnaik A.K., Flores E.F., Osorio F.A. (2008). Development of a porcine reproductive and respiratory syndrome virus differentiable (DIVA) strain through deletion of specific immunodominant epitopes. Vaccine.

[209] de Lima M., Pattnaik A.K., Flores E.F., Osorio F.A. (2006). Serologic marker candidates identified among B cell linear epitopes of Nsp2 and structural proteins of a North American strain of porcine reproductive and respiratory syndrome virus. Virology.

[210] Fang Y., Christopher-Hennings J., Brown E., Liu H., Chen Z., Lawson S.R., Breen R., Clement T., Gao X., Bao J. (2008). Development of genetic markers in the non-structural protein 2 region of a US type 1 porcine reproductive and respiratory syndrome virus: Implications for future recombinant marker vaccine development. J. Gen. Virol..

[211] Xu Y.Z., Zhou Y.J., Zhang S.R., Jiang Y.F., Tong W., Yu H., Tong G.Z. (2012). Stable expression of foreign gene in nonessential region of nonstructural protein 2 (nsp2) of porcine reproductive and respiratory syndrome virus: Applications for marker vaccine design. Vet. Microbiol..

[212] Zhao J., Duan H., Chen X., Ren B., Zhu Q., Ji P., Chang Y., Sun Y., Zhao Q. (2025). A serologic marker attenuated live vaccine protects piglets against highly pathogenic porcine reproductive and respiratory syndrome virus infection. Vet. Res..

[213] Li C., Liu Z., Chen K., Qian J., Hu Y., Fang S., Sun Z., Zhang C., Huang L., Zhang J. (2022). Efficacy of the Synergy Between Live-Attenuated and Inactivated PRRSV Vaccines Against a NADC30-Like Strain of Porcine Reproductive and Respiratory Syndrome Virus in 4-Week Piglets. Front. Vet. Sci..

[214] Renson P., Mahé S., Andraud M., Le Dimna M., Paboeuf F., Rose N., Bourry O. (2024). Effect of vaccination route (intradermal vs. intramuscular) against porcine reproductive and respiratory syndrome using a modified live vaccine on systemic and mucosal immune response and virus transmission in pigs. BMC Vet. Res..

[215] Liu J., Yan X., Wu W., Li Y., Xing S., Zhao S., Huang X., Zhao Q., Wen Y., Cao S. (2024). Evaluation of cross-protection of a reduced-dose PRRS MLV vaccine against the NADC30-like PRRSV challenge. Front. Vet. Sci..

[216] Yan X., Liu J., Yue F., Lin Y., Li Y., Wu W., Zhao S., Huang X., Zhao Q., Wen Y. (2024). Efficacy of a reduced-dosage PRRS MLV vaccine against a NADC34-like strain of porcine reproductive and respiratory syndrome virus. Front. Vet. Sci..

[217] Jayaraman S., Bauman T., Maschhoff A., Shull C., Li P., Magalhaes E., Trevisan G., Linhares D.C.L., Li C., Silva G.S. (2025). Causal inference study of PRRSV-MLV vaccine dosing effects on wean-to-finish performance during outbreaks. Front. Vet. Sci..

[218] Fiers J., Maes D., Cay A.B., Vandenbussche F., Mostin L., Parys A., Tignon M. (2024). PRRSV-Vaccinated, Seronegative Sows and Maternally Derived Antibodies (II): Impact on PRRSV-1 Vaccine Effectiveness and Challenge Outcomes in Piglets. Vaccines.

[219] Martín-Valls G.E., Mortensen P., Clilvert H., Li Y., Cortey M., Sno M., Barna T., Terré M., Guerra N., Mateu E. (2022). The use of a whole inactivated PRRS virus vaccine administered in sows and impact on maternally derived immunity and timing of PRRS virus infection in piglets. Vet. Rec. Open.

[220] Shuai W., Sun Q., Li J., Shen W., Zhu J., Guo Z., Luo M., Zeng Y., Wang J., Tong G. (2026). Needle-free intradermal delivery of a recombinant PRRSV-vectored CSFV E2 vaccine enhances humoral immunity and growth performance in piglets. Vaccine.

[221] Dwivedi V., Manickam C., Patterson R., Dodson K., Murtaugh M., Torrelles J.B., Schlesinger L.S., Renukaradhya G.J. (2011). Cross-protective immunity to porcine reproductive and respiratory syndrome virus by intranasal delivery of a live virus vaccine with a potent adjuvant. Vaccine.

[222] Opriessnig T., Rawal G., McKeen L., Filippsen Favaro P., Halbur P.G., Gauger P.C. (2021). Evaluation of the intranasal route for porcine reproductive and respiratory disease modified-live virus vaccination. Vaccine.

[223] Prapattanakullawong A., Sirisereewan C., Boonprakob R., Kamenkit O., Kesdangsakonwut S., Thanawongnuwech R., Woonwong Y., Nedumpun T. (2025). Comparative evaluation of PRRSV MLV vaccine efficacy administered via intranasal and intramuscular routes in early age of piglets. Vaccine.

[224] Li X., Galliher-Beckley A., Huang H., Sun X., Shi J. (2013). Peptide nanofiber hydrogel adjuvanted live virus vaccine enhances cross-protective immunity to porcine reproductive and respiratory syndrome virus. Vaccine.

[225] Charerntantanakul W., Pongjaroenkit S. (2018). Co-administration of saponin quil A and PRRSV-1 modified-live virus vaccine up-regulates gene expression of type I interferon-regulated gene, type I and II interferon, and inflammatory cytokines and reduces viremia in response to PRRSV-2 challenge. Vet. Immunol. Immunopathol..

[226] Cao Q.M., Ni Y.Y., Cao D., Tian D., Yugo D.M., Heffron C.L., Overend C., Subramaniam S., Rogers A.J., Catanzaro N. (2018). Recombinant Porcine Reproductive and Respiratory Syndrome Virus Expressing Membrane-Bound Interleukin-15 as an Immunomodulatory Adjuvant Enhances NK and γδ T Cell Responses and Confers Heterologous Protection. J. Virol..

[227] Peng J., Wang J., Wu J., Du Y., Li J., Guo Z., Yu J., Xu S., Zhang Y., Sun W. (2013). Positive inductive effect of swine interleukin-4 on immune responses elicited by modified live porcine reproductive and respiratory syndrome virus (PRRSV) vaccine. Viral Immunol..

[228] Corzo C.A., Mondaca E., Wayne S., Torremorell M., Dee S., Davies P., Morrison R.B. (2010). Control and elimination of porcine reproductive and respiratory syndrome virus. Virus Res..

[229] Jeong J., Aly S.S., Cano J.P., Polson D., Kass P.H., Perez A.M. (2014). Stochastic model of porcine reproductive and respiratory syndrome virus control strategies on a swine farm in the United States. Am. J. Vet. Res..

[230] Amadori M., Listorti V., Razzuoli E. (2021). Reappraisal of PRRS Immune Control Strategies: The Way Forward. Pathogens.

[231] Assavacheep P., Thanawongnuwech R. (2022). Porcine respiratory disease complex: Dynamics of polymicrobial infections and management strategies after the introduction of the African swine fever. Front. Vet. Sci..

[232] Berton P., Normand V., Martineau G.P., Bouchet F., Lebret A., Waret-Szkuta A. (2017). Evaluation of porcine reproductive and respiratory syndrome stabilization protocols in 23 French Farrow-to-finish farms located in a high-density swine area. Porc. Health Manag..

[233] Toman M., Celer V., Kavanová L., Levá L., Frolichova J., Ondráčková P., Kudláčková H., Nechvátalová K., Salat J., Faldyna M. (2019). Dynamics and Differences in Systemic and Local Immune Responses After Vaccination With Inactivated and Live Commercial Vaccines and Subsequent Subclinical Infection With PRRS Virus. Front. Immunol..

[234] Pandey M.K., Rajukumar K., Senthilkumar D., Kombiah S., Singh F., Venkatesh G., Kumar M., Shrivas S., Shrivastava D., Singh V.P. (2023). Evaluation of dynamics of immune responses and protective efficacy in piglets immunized with an inactivated porcine reproductive and respiratory syndrome vaccine candidate. Vaccine.

[235] Delrue I., Delputte P.L., Nauwynck H.J. (2009). Assessing the functionality of viral entry-associated domains of porcine reproductive and respiratory syndrome virus during inactivation procedures, a potential tool to optimize inactivated vaccines. Vet. Res..

[236] Renukaradhya G.J., Meng X.J., Calvert J.G., Roof M., Lager K.M. (2015). Inactivated and subunit vaccines against porcine reproductive and respiratory syndrome: Current status and future direction. Vaccine.

[237] Geldhof M.F., Van Breedam W., De Jong E., Lopez Rodriguez A., Karniychuk U.U., Vanhee M., Van Doorsselaere J., Maes D., Nauwynck H.J. (2013). Antibody response and maternal immunity upon boosting PRRSV-immune sows with experimental farm-specific and commercial PRRSV vaccines. Vet. Microbiol..

[238] Papatsiros V.G., Alexopoulos C., Kritas S.K., Koptopoulos G., Nauwynck H.J., Pensaert M.B., Kyriakis S.C. (2006). Long-term administration of a commercial porcine reproductive and respiratory syndrome virus (PRRSV)-inactivated vaccine in PRRSV-endemically infected sows. J. Vet. Med. B Infect. Dis. Vet. Public Health.

[239] Charerntantanakul W. (2009). Adjuvants for porcine reproductive and respiratory syndrome virus vaccines. Vet. Immunol. Immunopathol..

[240] Dwivedi V., Manickam C., Binjawadagi B., Renukaradhya G.J. (2013). PLGA nanoparticle entrapped killed porcine reproductive and respiratory syndrome virus vaccine helps in viral clearance in pigs. Vet. Microbiol..

[241] Chen R., Liu B., Zhang X., Qin M., Dong J., Gu G., Wu C., Wang J., Nan Y. (2022). A porcine reproductive and respiratory syndrome virus (PRRSV)-specific IgM as a novel adjuvant for an inactivated PRRSV vaccine improves protection efficiency and enhances cell-mediated immunity against heterologous PRRSV challenge. Vet. Res..

[242] Zhang H., Luo Q., He Y., Zheng Y., Sha H., Li G., Kong W., Liao J., Zhao M. (2023). Research Progress on the Development of Porcine Reproductive and Respiratory Syndrome Vaccines. Vet. Sci..

[243] Chia M.Y., Hsiao S.H., Chan H.T., Do Y.Y., Huang P.L., Chang H.W., Tsai Y.C., Lin C.M., Pang V.F., Jeng C.R. (2010). Immunogenicity of recombinant GP5 protein of porcine reproductive and respiratory syndrome virus expressed in tobacco plant. Vet. Immunol. Immunopathol..

[244] Hu J., Ni Y., Dryman B.A., Meng X.J., Zhang C. (2012). Immunogenicity study of plant-made oral subunit vaccine against porcine reproductive and respiratory syndrome virus (PRRSV). Vaccine.

[245] Du L., Li B., Pang F., Yu Z., Xu X., Fan B., Tan Y., He K., Huang K. (2017). Porcine GPX1 enhances GP5-based DNA vaccination against porcine reproductive and respiratory syndrome virus. Vet. Immunol. Immunopathol..

[246] Uribe-Campero L., Monroy-García A., Durán-Meza A.L., Villagrana-Escareño M.V., Ruíz-García J., Hernández J., Núñez-Palenius H.G., Gómez-Lim M.A. (2015). Plant-based porcine reproductive and respiratory syndrome virus VLPs induce an immune response in mice. Res. Vet. Sci..

[247] Murthy A.M., Ni Y., Meng X., Zhang C. (2015). Production and evaluation of virus-like particles displaying immunogenic epitopes of porcine reproductive and respiratory syndrome virus (PRRSV). Int. J. Mol. Sci..

[248] Piron R., De Koker S., De Paepe A., Goossens J., Grooten J., Nauwynck H., Depicker A. (2014). Boosting In Planta Production of Antigens Derived from the Porcine Reproductive and Respiratory Syndrome Virus (PRRSV) and Subsequent Evaluation of Their Immunogenicity. PLoS ONE.

[249] Plana Duran J., Climent I., Sarraseca J., Urniza A., Cortés E., Vela C., Casal J.I. (1997). Baculovirus expression of proteins of porcine reproductive and respiratory syndrome virus strain Olot/91. Involvement of ORF3 and ORF5 proteins in protection. Virus Genes.

[250] Chia M.Y., Hsiao S.H., Chan H.T., Do Y.Y., Huang P.L., Chang H.W., Tsai Y.C., Lin C.M., Pang V.F., Jeng C.R. (2011). Evaluation of the immunogenicity of a transgenic tobacco plant expressing the recombinant fusion protein of GP5 of porcine reproductive and respiratory syndrome virus and B subunit of Escherichia coli heat-labile enterotoxin in pigs. Vet. Immunol. Immunopathol..

[251] Nam H.M., Chae K.S., Song Y.J., Lee N.H., Lee J.B., Park S.Y., Song C.S., Seo K.H., Kang S.M., Kim M.C. (2013). Immune responses in mice vaccinated with virus-like particles composed of the GP5 and M proteins of porcine reproductive and respiratory syndrome virus. Arch. Virol..

[252] Jiang W., Jiang P., Wang X., Li Y., Du Y., Wang X. (2008). Enhanced immune responses of mice inoculated recombinant adenoviruses expressing GP5 by fusion with GP3 and/or GP4 of PRRS virus. Virus Res..

[253] Zheng Q., Chen D., Li P., Bi Z., Cao R., Zhou B., Chen P. (2007). Co-expressing GP5 and M proteins under different promoters in recombinant modified vaccinia virus ankara (rMVA)-based vaccine vector enhanced the humoral and cellular immune responses of porcine reproductive and respiratory syndrome virus (PRRSV). Virus Genes.

[254] Zhao G., Zhang J., Sun W., Xie C., Zhang H., Gao Y., Wen S., Ha Z., Nan F., Zhu X. (2022). Immunological evaluation of recombination PRRSV GP3 and GP5 DNA vaccines in vivo. Front. Cell. Infect. Microbiol..

[255] Ren J.Q., Sun W.C., Lu H.J., Wen S.B., Jing J., Yan F.L., Liu H., Liu C.X., Xiao P.P., Chen X. (2014). Construction and immunogenicity of a DNA vaccine coexpressing GP3 and GP5 of genotype-I porcine reproductive and respiratory syndrome virus. BMC Vet. Res..

[256] Eck M., Durán M.G., Ricklin M.E., Locher S., Sarraseca J., Rodríguez M.J., McCullough K.C., Summerfield A., Zimmer G., Ruggli N. (2016). Virus replicon particles expressing porcine reproductive and respiratory syndrome virus proteins elicit immune priming but do not confer protection from viremia in pigs. Vet. Res..

[257] Roques E., Girard A., St-Louis M.C., Massie B., Gagnon C.A., Lessard M., Archambault D. (2013). Immunogenic and protective properties of GP5 and M structural proteins of porcine reproductive and respiratory syndrome virus expressed from replicating but nondisseminating adenovectors. Vet. Res..

[258] de Brito R.C.F., Holtham K., Roser J., Saunders J.E., Wezel Y., Henderson S., Mauch T., Sanz-Bernardo B., Frossard J.P., Bernard M. (2023). An attenuated herpesvirus vectored vaccine candidate induces T cell responses against highly conserved porcine reproductive and respiratory syndrome virus M and NSP5 proteins that are unable to control infection. Front. Immunol..

[259] Wang S., Fang L., Fan H., Jiang Y., Pan Y., Luo R., Zhao Q., Chen H., Xiao S. (2007). Construction and immunogenicity of pseudotype baculovirus expressing GP5 and M protein of porcine reproductive and respiratory syndrome virus. Vaccine.

[260] Jiang W., Jiang P., Li Y., Wang X., Du Y. (2007). Analysis of immunogenicity of minor envelope protein GP3 of porcine reproductive and respiratory syndrome virus in mice. Virus Genes.

[261] Charerntantanakul W. (2012). Porcine reproductive and respiratory syndrome virus vaccines: Immunogenicity, efficacy and safety aspects. World J. Virol..

[262] Xie C.Z., Zhang P., Wang Z., Tao Y.M., Cui Z.D., Nan F.L., Zhang F.C., Ren Y.X., Zhang H., Lu H.J. (2024). Construction and immunological evaluation of recombinant adenovirus vaccines of new novel NADC34-PRRSV strains in pigs. Front. Vet. Sci..

[263] Rompato G., Ling E., Chen Z., Van Kruiningen H., Garmendia A.E. (2006). Positive inductive effect of IL-2 on virus-specific cellular responses elicited by a PRRSV-ORF7 DNA vaccine in swine. Vet. Immunol. Immunopathol..

[264] Tang D., Liu J., Li C., Zhang H., Ma P., Luo X., Zeng Z., Hong N., Liu X., Wang B. (2014). Positive effects of porcine IL-2 and IL-4 on virus-specific immune responses induced by the porcine reproductive and respiratory syndrome virus (PRRSV) ORF5 DNA vaccine in swine. J. Vet. Sci..

[265] Zhang X., Wang X., Mu L., Ding Z. (2012). Immune responses in pigs induced by recombinant DNA vaccine co-expressing swine IL-18 and membrane protein of porcine reproductive and respiratory syndrome virus. Int. J. Mol. Sci..

[266] Zhang X., Li G., Gao L., Mu L., Zhang L., Cong Y., Ding Z. (2013). Positive inductive effect of IL-18 on virus-specific immune responses induced by PRRSV-GP5 DNA vaccine in swine. Res. Vet. Sci..

[267] Du Y., Qi J., Lu Y., Wu J., Yoo D., Liu X., Zhang X., Li J., Sun W., Cong X. (2012). Evaluation of a DNA vaccine candidate co-expressing GP3 and GP5 of porcine reproductive and respiratory syndrome virus (PRRSV) with interferon α/γ in immediate and long-lasting protection against HP-PRRSV challenge. Virus Genes.

[268] Fang L., Jiang Y., Xiao S., Niu C., Zhang H., Chen H. (2006). Enhanced immunogenicity of the modified GP5 of porcine reproductive and respiratory syndrome virus. Virus Genes.

[269] Popescu L.N., Trible B.R., Chen N., Rowland R.R.R. (2017). GP5 of porcine reproductive and respiratory syndrome virus (PRRSV) as a target for homologous and broadly neutralizing antibodies. Vet. Microbiol..

[270] Van Breedam W., Van Gorp H., Zhang J.Q., Crocker P.R., Delputte P.L., Nauwynck H.J. (2010). The M/GP(5) glycoprotein complex of porcine reproductive and respiratory syndrome virus binds the sialoadhesin receptor in a sialic acid-dependent manner. PLoS Pathog..

[271] Rong Z., Jiao M., Tian L., Wang X., Zhang M., Zhang C., Liu S., Wei L., Chen H., Li X. (2025). PRRSV vector vaccine based on VEEV-VSVG recombinant replicon elicits efficient immune responses in piglets. Vaccine.

[272] Tian L., Lin Z., Pan M., Wen Y., Lu M., Liu S., Zhang C., Rong Z., Ma J., Zhou R. (2026). A multicomponent Fc-fused subunit vaccine induces cross-protective immunity against porcine reproductive and respiratory syndrome virus QYYZ-like and NADC30-like strains. Vet. Microbiol..

[273] Sun Y., Gao Y., Su T., Zhang L., Zhou H., Zhang J., Sun H., Bai J., Jiang P. (2025). Nanoparticle Vaccine Triggers Interferon-Gamma Production and Confers Protective Immunity against Porcine Reproductive and Respiratory Syndrome Virus. ACS Nano.

[274] Pardi N., Hogan M.J., Porter F.W., Weissman D. (2018). mRNA vaccines—A new era in vaccinology. Nat. Rev. Drug Discov..

[275] Zhou L., Wubshet A.K., Zhang J., Hou S., Yao K., Zhao Q., Dai J., Liu Y., Ding Y., Zhang J. (2024). The mRNA Vaccine Expressing Single and Fused Structural Proteins of Porcine Reproductive and Respiratory Syndrome Induces Strong Cellular and Humoral Immune Responses in BalB/C Mice. Viruses.

[276] Sahin U., Karikó K., Türeci Ö. (2014). mRNA-based therapeutics--developing a new class of drugs. Nat. Rev. Drug Discov..

[277] Xiao Y., Tang Z., Huang X., Chen W., Zhou J., Liu H., Liu C., Kong N., Tao W. (2022). Emerging mRNA technologies: Delivery strategies and biomedical applications. Chem. Soc. Rev..

[278] Seo S.H., Song M.K. (2025). Advancements and challenges in next-generation mRNA vaccine manufacturing systems. Clin. Exp. Vaccine Res..

[279] Zhou H., Sun Y., Zhang L., Liang X., Bai J., Gao Y., Jiang P. (2026). An mRNA vaccine encoding multiple envelope glycoproteins of porcine reproductive and respiratory syndrome virus confers efficient protection against NADC30-like strains. Int. J. Biol. Macromol..

[280] Liu Y., Li Y., Hu Q. (2023). Advances in saRNA Vaccine Research against Emerging/Re-Emerging Viruses. Vaccines.

[281] Blakney A.K., Ip S., Geall A.J. (2021). An Update on Self-Amplifying mRNA Vaccine Development. Vaccines.

[282] Blakney A.K., McKay P.F., Hu K., Samnuan K., Jain N., Brown A., Thomas A., Rogers P., Polra K., Sallah H. (2021). Polymeric and lipid nanoparticles for delivery of self-amplifying RNA vaccines. J. Control. Release.

[283] Tanaka H., Sakurai Y., Akita H. (2022). Lipid nanoparticles for mRNA delivery. Drug Deliv. Syst..

[284] Liu H., Ma Y., Hou F., Teng Z., Jiang L., Qiao L., Mehdi M.M., Yin S., Guo H., Sun S. (2026). Combination immunization with mRNAs encoding PRRSV antigens enhances immune responses and confers protective immunity against highly pathogenic PRRSV in piglets. Virology.

[285] Liu J., Ni S., Lv Y., Tong Z., Liu P., Zong X., Chen G., Zeng Y., Wang C., Tan C. (2025). Protective Efficacy of an mRNA Vaccine Against HP-PRRSV Challenge in Piglets. Microorganisms.

[286] Pardi N., Krammer F. (2024). mRNA vaccines for infectious diseases—Advances, challenges and opportunities. Nat. Rev. Drug Discov..

[287] Khan M., Taira O., Shi X., Wang H., Cai X. (2025). mRNA vaccines for porcine reproductive and respiratory syndrome: A new paradigm in swine health. Biochem. Biophys. Res. Commun..

[288] Amona F.M., Pang Y., Gong X., Wang Y., Fang X., Zhang C., Chen X. (2024). Mechanism of PRRSV infection and antiviral role of polyphenols. Virulence.

[289] Du T., Nan Y., Xiao S., Zhao Q., Zhou E.M. (2017). Antiviral Strategies against PRRSV Infection. Trends Microbiol..

[290] Bello-Onaghise G., Wang G., Han X., Nsabimana E., Cui W., Yu F., Zhang Y., Wang L., Li Z., Cai X. (2020). Antiviral Strategies of Chinese Herbal Medicine Against PRRSV Infection. Front. Microbiol..

[291] Zhang M., Qian B., Kunec D., Veit M. (2025). Development of GFP-expressing infectious clones for PRRSV using TAR cloning for antiviral drug screening. npj Viruses.

[292] Xie J., Zhou H., Cui J., Chen Y., Zhang M., Deng S., Zhou P., Su S., Zhang G. (2014). Inhibition of porcine reproductive and respiratory syndrome virus by specific siRNA targeting Nsp9 gene. Infect. Genet. Evol..

[293] Gao L., Zhang W., Sun Y., Yang Q., Ren J., Liu J., Wang H., Feng W.H. (2013). Cryptoporus volvatus extract inhibits porcine reproductive and respiratory syndrome virus (PRRSV) in vitro and in vivo. PLoS ONE.

[294] Liu H., Wang Y., Duan H., Zhang A., Liang C., Gao J., Zhang C., Huang B., Li Q., Li N. (2015). An intracellularly expressed Nsp9-specific nanobody in MARC-145 cells inhibits porcine reproductive and respiratory syndrome virus replication. Vet. Microbiol..

[295] Wang L., Zhang L., Huang B., Li K., Hou G., Zhao Q., Wu C., Nan Y., Du T., Mu Y. (2019). A Nanobody Targeting Viral Nonstructural Protein 9 Inhibits Porcine Reproductive and Respiratory Syndrome Virus Replication. J. Virol..

[296] Zhang L., Wang L., Cao S., Lv H., Huang J., Zhang G., Tabynov K., Zhao Q., Zhou E.M. (2021). Nanobody Nb6 fused with porcine IgG Fc as the delivering tag to inhibit porcine reproductive and respiratory syndrome virus replication in porcine alveolar macrophages. Vet. Res..

[297] Shi Y., Lei Y., Ye G., Sun L., Fang L., Xiao S., Fu Z.F., Yin P., Song Y., Peng G. (2018). Identification of two antiviral inhibitors targeting 3C-like serine/3C-like protease of porcine reproductive and respiratory syndrome virus and porcine epidemic diarrhea virus. Vet. Microbiol..

[298] Wang Z.Z., Xiao C.X., Huang W.L., Hu Y., Zhang H.T., Liu Z., Peng S.H., Wei Z. (2025). Discovery of Flavonol derivatives as porcine reproductive and respiratory syndrome virus inhibitors. Bioorg. Med. Chem. Lett..

[299] Wei L., Liu Z., Zhang H., Wang G., Weng J., Hu L., Wang W., Li S., Ming K., Wei Z. (2026). Shikonin covalently binding to cysteine to inhibits the cleavage activity of the 3C-like serine main protease (Nsp4) of PRRSV-2 and suppresses viral replication in Marc-145 cells. npj Vet. Sci..

[300] Pathak R.K., Seo Y.J., Kim J.M. (2022). Structural insights into inhibition of PRRSV Nsp4 revealed by structure-based virtual screening, molecular dynamics, and MM-PBSA studies. J. Biol. Eng..

[301] Wells K.D., Bardot R., Whitworth K.M., Trible B.R., Fang Y., Mileham A., Kerrigan M.A., Samuel M.S., Prather R.S., Rowland R.R.R. (2017). Replacement of Porcine CD163 Scavenger Receptor Cysteine-Rich Domain 5 with a CD163-Like Homolog Confers Resistance of Pigs to Genotype 1 but Not Genotype 2 Porcine Reproductive and Respiratory Syndrome Virus. J. Virol..

[302] Buechler C., Eisinger K., Krautbauer S. (2013). Diagnostic and prognostic potential of the macrophage specific receptor CD163 in inflammatory diseases. Inflamm. Allergy Drug Targets.

[303] Guo C., Wang M., Zhu Z., He S., Liu H., Liu X., Shi X., Tang T., Yu P., Zeng J. (2019). Highly Efficient Generation of Pigs Harboring a Partial Deletion of the CD163 SRCR5 Domain, Which Are Fully Resistant to Porcine Reproductive and Respiratory Syndrome Virus 2 Infection. Front. Immunol..

[304] Burkard C., Opriessnig T., Mileham A.J., Stadejek T., Ait-Ali T., Lillico S.G., Whitelaw C.B.A., Archibald A.L. (2018). Pigs Lacking the Scavenger Receptor Cysteine-Rich Domain 5 of CD163 Are Resistant to Porcine Reproductive and Respiratory Syndrome Virus 1 Infection. J. Virol..

[305] Burkard C., Lillico S.G., Reid E., Jackson B., Mileham A.J., Ait-Ali T., Whitelaw C.B., Archibald A.L. (2017). Precision engineering for PRRSV resistance in pigs: Macrophages from genome edited pigs lacking CD163 SRCR5 domain are fully resistant to both PRRSV genotypes while maintaining biological function. PLoS Pathog..

[306] Nesbitt C., Galina Pantoja L., Beaton B., Chen C.Y., Culbertson M., Harms P., Holl J., Sosnicki A., Reddy S., Rotolo M. (2024). Pigs lacking the SRCR5 domain of CD163 protein demonstrate heritable resistance to the PRRS virus and no changes in animal performance from birth to maturity. Front. Genome Ed..

[307] Salgado B., Rivas R.B., Pinto D., Sonstegard T.S., Carlson D.F., Martins K., Bostrom J.R., Sinebo Y., Rowland R.R.R., Brandariz-Nuñez A. (2024). Genetically modified pigs lacking CD163 PSTII-domain-coding exon 13 are completely resistant to PRRSV infection. Antivir. Res..

[308] Liu Y., Yang L., Xiang H.Y., Niu M., Deng J.C., Li X.Y., Hao W.J., Ou-Yang H.S., Liu T.Y., Tang X.C. (2024). Genetically modified pigs with CD163 point mutation are resistant to HP-PRRSV infection. Zool. Res..

[309] Xu H., Liu Z., Zheng S., Han G., He F. (2020). CD163 Antibodies Inhibit PRRSV Infection via Receptor Blocking and Transcription Suppression. Vaccines.

[310] Wang H., Shen L., Chen J., Liu X., Tan T., Hu Y., Bai X., Li Y., Tian K., Li N. (2019). Deletion of CD163 Exon 7 Confers Resistance to Highly Pathogenic Porcine Reproductive and Respiratory Viruses on Pigs. Int. J. Biol. Sci..

[311] Durazo-Martínez K., Chaudhari J., Sherry L.M., Webster D.A., Martins K., Bostrom J.R., Carlson D.F., Sonstegard T.S., Vu H.L.X. (2025). Modification of the splice acceptor in CD163 exon 7 of pigs is insufficient to confer resistance to PRRSV. Vet. Microbiol..

[312] Burger B.T., Beaton B.P., Campbell M.A., Brett B.T., Rohrer M.S., Plummer S., Barnes D., Jiang K., Naswa S., Lange J. (2024). Generation of a Commercial-Scale Founder Population of Porcine Reproductive and Respiratory Syndrome Virus Resistant Pigs Using CRISPR-Cas. CRISPR J..

[313] Mehravar M., Shirazi A., Nazari M., Banan M. (2019). Mosaicism in CRISPR/Cas9-mediated genome editing. Dev. Biol..

[314] Li Z., Lan J., Shi X., Lu T., Hu X., Liu X., Chen Y., He Z. (2024). Whole-Genome Sequencing Reveals Rare Off-Target Mutations in MC1R-Edited Pigs Generated by Using CRISPR-Cas9 and Somatic Cell Nuclear Transfer. CRISPR J..

[315] Cigan A.M., Knap P. (2022). Technical considerations towards commercialization of porcine respiratory and reproductive syndrome (PRRS) virus resistant pigs. CABI Agric. Biosci..

[316] Van Eenennaam A.L., De Figueiredo Silva F., Trott J.F., Zilberman D. (2021). Genetic Engineering of Livestock: The Opportunity Cost of Regulatory Delay. Annu. Rev. Anim. Biosci..

[317] Martin-Collado D., Byrne T.J., Crowley J.J., Kirk T., Ripoll G., Whitelaw C.B.A. (2022). Gene-Edited Meat: Disentangling Consumers’ Attitudes and Potential Purchase Behavior. Front. Nutr..

[318] Ledesma A.V., Van Eenennaam A.L. (2024). Global status of gene edited animals for agricultural applications. Vet. J..

[319] Zhu J., He X., Bernard D., Shen J., Su Y., Wolek A., Issacs B., Mishra N., Tian X., Garmendia A. (2023). Identification of New Compounds against PRRSV Infection by Directly Targeting CD163. J. Virol..

[320] Han G., Yang H., Xu H., Zheng S., Li Y., Liu Z., He F. (2022). Broad antiviral peptides against PRRSV based on novel linear epitopes on porcine CD163. Int. J. Biol. Macromol..

[321] Yang H., Sun M., Qiu H., Xu H., Deng Z., Gu H., Wang N., Du L., Shi F., Zhou J. (2024). Nanobody peptide conjugate: A novel CD163 based broad neutralizing strategy against porcine reproductive and respiratory syndrome virus. J. Nanobiotechnology.

[322] Cui Z., Liu J., Xie C., Wang T., Sun P., Wang J., Li J., Li G., Qiu J., Zhang Y. (2024). High-throughput screening unveils nitazoxanide as a potent PRRSV inhibitor by targeting NMRAL1. Nat. Commun..

[323] Zhang X., Guo C. (2022). Recent advances in inhibition of porcine reproductive and respiratory syndrome virus through targeting CD163. Front. Microbiol..

[324] Magalhães E.S., Zimmerman J.J., Holtkamp D.J., Classen D.M., Groth D.D., Glowzenski L., Philips R., Silva G.S., Linhares D.C.L. (2021). Next Generation of Voluntary PRRS Virus Regional Control Programs. Front. Vet. Sci..

[325] Teixeira Costa C., Lebret A., Bougeard S., Rose N. (2025). PRRS management by European swine veterinarians: A descriptive insight into practices and profiles. Porc. Health Manag..

[326] Neumann E.J., Kliebenstein J.B., Johnson C.D., Mabry J.W., Bush E.J., Seitzinger A.H., Green A.L., Zimmerman J.J. (2005). Assessment of the economic impact of porcine reproductive and respiratory syndrome on swine production in the United States. J. Am. Vet. Med. Assoc..

